# Applications of cell resealing to reconstitute microRNA loading to extracellular vesicles

**DOI:** 10.1038/s41598-021-82452-5

**Published:** 2021-02-03

**Authors:** Yuki Sonoda, Fumi Kano, Masayuki Murata

**Affiliations:** 1 Department of Life Sciences, Graduate School of Arts and Sciences, The University of Tokyo, 3-8-1 Komaba, Meguro-ku, Tokyo, 153-8902 Japan; 2 Cell Biology Center, Institute of Innovative Research, Tokyo Institute of Technology, 4259 Nagatsuta, Midori-ku, Yokohama, Kanagawa 226-8503 Japan

**Keywords:** Biochemistry, RNA-binding proteins, miRNAs, Membrane trafficking, Exocytosis, Multivesicular bodies, Analytical biochemistry, Cytological techniques, Fluorescence imaging, Confocal microscopy, Super-resolution microscopy, Nanoparticles

## Abstract

MicroRNAs (miRNAs) are cargo carried by extracellular vesicles (EVs) and are associated with cell–cell interactions. The response to the cellular environment, such as disease states, genetic/metabolic changes, or differences in cell type, highly regulates cargo sorting to EVs. However, morphological features during EV formation and secretion involving miRNA loading are unknown. This study developed a new method of EV loading using cell resealing and reconstituted the elementary miRNA-loading processes. Morphology, secretory response, and cellular uptake ability of EVs obtained from intact and resealed HeLa cells were comparable. Exogenously added soluble factors were introduced into multivesicular endosomes (MVEs) and their subsequent secretion to the extracellular region occurred in resealed HeLa cells. In addition, miRNA transport to MVEs and miRNA encapsulation to EVs followed a distinct pathway regulated by RNA-binding proteins, such as Argonaute and Y-box binding protein 1, depending on miRNA types. Our cell-resealing system can analyze disease-specific EVs derived from disease model cells, where pathological cytosol is introduced into cells. Thus, EV formation in resealed cells can be used not only to create a reconstitution system to give mechanistic insight into EV encapsulation but also for applications such as loading various molecules into EVs and identifying disease-specific EV markers.

## Introduction

Extracellular vesicles (EVs), including microvesicles, apoptotic bodies, and exosomes, are released by a cell in an environmentally dependent manner. Microvesicles and apoptotic bodies are generated by plasma membrane budding and detachment, while canonical exosomes are generated by more complicated and regulated processes^1,2^. First, late endosomal membranes are invaginated to form multiple intraluminal vesicles (ILVs) inside multivesicular endosomes (MVEs). Second, MVEs fuse with the plasma membrane, releasing ILVs into the extracellular region. These ILVs with a diameter of ~ 100 nm are referred to as “exosomes” in this study. EVs contain various molecules, such as lipids, proteins, RNA, and DNA, derived from donor cells. After EVs are released from cells, they are uptaken into recipient cells via direct membrane fusion with the plasma membrane or via endocytosis^3,4^. The cargoes carried by EVs incorporated into recipient cells can change the biochemical environments of the recipient cells by affecting their signal transduction pathways, metabolic pathways, and genetic modulation^3^, causing tumor progression, metabolic disorders, neurological disorders, aging, and infections^5^. Various EV functions depend on their production efficiencies, membranous components, and loaded cargoes. Therefore, understanding the mechanisms underlying EV formation and secretion is crucial to using EVs for therapeutic research and determining the pathogenesis of diseases via intercellular communications by EVs.

MicroRNAs (miRNAs), a type of EV cargo, inhibit a wide range of proteins and rearrange the cellular environment. After transcription and processing by Drosha form a precursor miRNA (pre-miRNA) in the nucleus, the pre-miRNA is transported to the cytoplasm, where Dicer, an RNase III family enzyme, cleaves the loop part of the pre-miRNA to form a double-stranded miRNA intermediate^6,7^. Argonaute (Ago), which has an RNase H-like PIWI domain, binds to the double-stranded miRNA, forming the RNA-induced silencing complex (RISC), which has an RNA-based silencing function^8,9^. Many studies have reported the molecular mechanisms underlying EV formation, including specific miRNA in/from cells^10^. Various molecules, such as Rab families, the endosomal sorting complexes required for transport (ESCRT) machinery, RNA-binding proteins (RBPs), and the soluble NSF attachment protein (SNAP) receptor (SNARE), are involved in EV formation, sorting, and secretion. The component of the RISC and trans-activation-responsive RNA-binding protein (TARPB) that interacts with Argonaute 2 (Ago2)^11^ is also involved in miRNA-loaded EV formation.

Studies have also investigated intracellular functions of the identified proteins and genes involved in EV formation in living cells via knockout/knockdown of specific genes, expression of dominant active/negative proteins, and utilization of modified miRNA^10,12,13^. In addition, the cell-free reconstitution of cargo sorting to isolated MVEs is especially interesting for determining the distinct sorting mechanisms for distinct EVs populations derived from cells with different phenotypes^14–18^. Recent advances in super-resolution microscopy allow us to observe the detailed structure of MVEs and the dynamic behavior of DNA or EV-related proteins in MVEs in living cells^13,19^. Therefore, a mechanistic understanding of EV formation in/from cells has grown extensively.

We have been working on membrane traffic and organelle dynamics using semi-intact cells and evolutionary resealed cell coupling using quantitative image-based microscopic analysis^20^. Semi-intact cells have plasma membranes that are permeabilized with the streptococcal toxin streptolysin O (SLO). SLO binds to cholesterol in the plasma membrane and oligomerizes to form ~ 30-nm-diameter pores. SLO-mediated pores allow various molecules, such as proteins, nucleotides, and membrane-impermeable small molecules, to enter cells. Therefore, a semi-intact cell system enables the exchange of cytosol to modified cytosol that was prepared from other cells/tissues, enabling us to reconstitute various intracellular phenomena, such as morphological changes in organelles during mitosis, vesicular transport, and organelle-specific protein targeting^21,22^. As previously reported^23^, pores are repaired in a Ca^2+^-dependent manner and SLOs are removed from cells through secretion microvesicles, which make semi-intact cells intact again. These cells are called resealed cells. Resealed cells containing modified cytosol are used as a cellular-type “test tube,” in which we can reconstitute modified cytosol-dependent intracellular events and analyze the underlying mechanisms biochemically or morphologically (by using microscopic analysis)^24,25^. In addition, introducing fluorescently labeled miRNAs into cells directly by cell resealing allows, using confocal microscopy or super-resolution microscopy, the visualization and measurement of exogenously added cytosol-dependent translocation, subcellular localization, co-localization with proteins, and dynamic behaviors of miRNA quantitatively in resealed cells. Especially, various modifications of exogenously added cytosol, such as immunodepletion of proteins, addition of dominant active/negative proteins, or addition of function-blocking antibodies, allow us to investigate specific functions of proteins of interest in resealed cells. Therefore, we tried to apply cell-resealing techniques to investigate the molecular mechanisms of EV formation in cells.

This study reproduced exogenously added cytosol-dependent EV formation using cell resealing. By using the system coupled with quantitative image-based microscopic analysis, we analyzed the dynamic behavior, subcellular localization, and co-localization of exogenously added, fluorescently labeled miRNA with CD63-positive MVEs or ILVs quantitatively in resealed HeLa cells using confocal microscopy or super-resolution microscopy. Finally, using morphometric analysis, we determined Ago-/Y-box binding protein 1 (YBX1)-mediated sorting/recruiting mechanisms for EV formation, depending on the miRNA species in resealed HeLa cells.

## Results

### Morphological characterization of EVs derived from resealed HeLa cells

**Figure 1:**
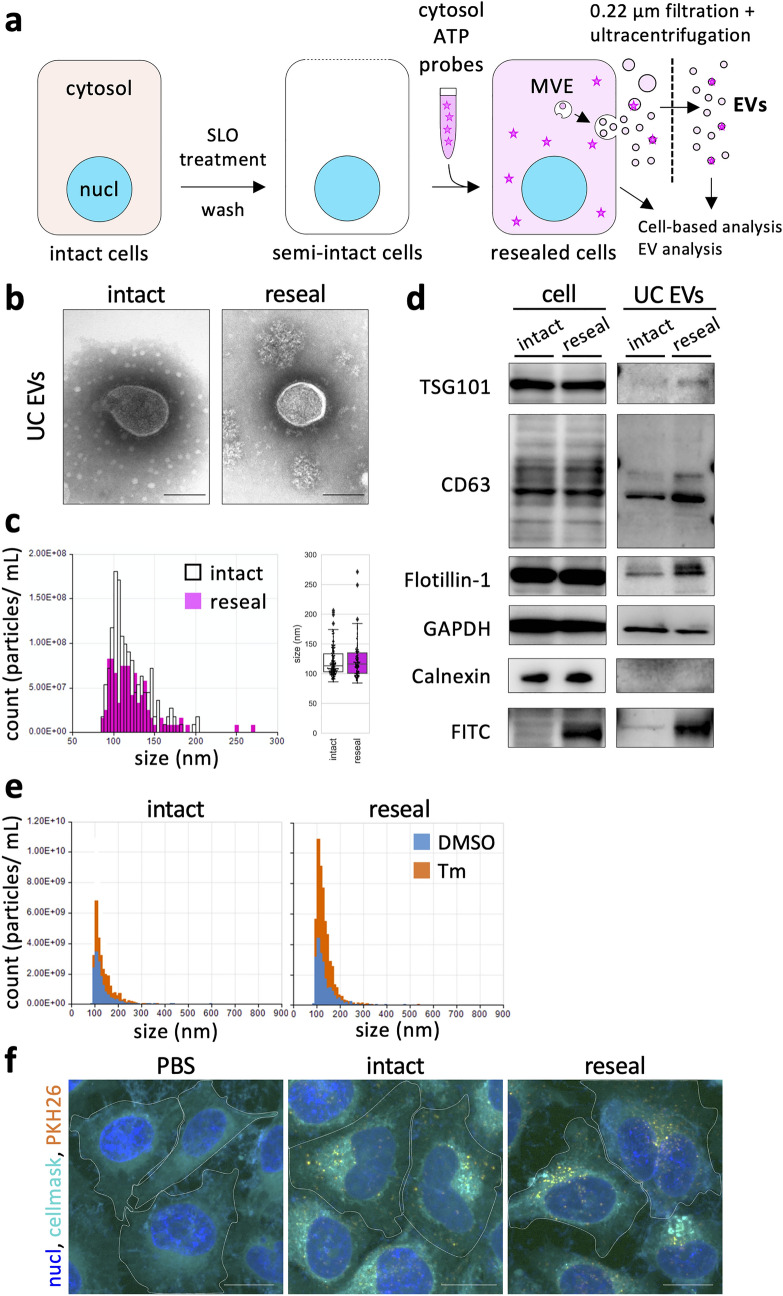
Morphological and biochemical characterization of EVs derived from resealed HeLa cells. (**a**) Reconstitution scheme of extracellular vesicle (EV) formation and secretion using HeLa cell resealing. Semi-intact HeLa cells were prepared by permeabilizing HeLa cells with SLO treatment. Resealed cells were prepared, as described in Methods, and were incubated under various conditions, and then culture media were collected. The resulting EV fraction was obtained by ultracentrifugation and subjected to various experiments. (**b**) Resealed HeLa cells were incubated in a 5% CO_2_ incubator for 120 min at 37 °C (post-incubation), and the medium was changed with a fresh one. The cells were further incubated in a 5% CO_2_ incubator for 48 h at 37 °C, and EVs were prepared, as described in (**a**), and subjected to negative-stained electron microscopy (EM) analysis. Scale bar = 100 nm. (**c**) Tunable resistive pulse sensing (TRPS) analysis was performed for EVs. Diluted EVs normalized by the number of cells were loaded to an NP200 nanopore. The minimum number of events recorded was 111 particles/measurement. The mean RMS noise was 4.4 ± 0.5 pA. The membrane stretch range used was 47.97 nm, the voltage used was 0.68 V, and the current range used was 91.6–94.3 nA. (**d**) Western blotting (WB) analysis was performed for whole cells and EVs obtained by ultracentrifugation (UC EVs). After introducing cytosol containing 100 µg/mL of FITC-BSA, we obtained EVs and whole-cell lysates (− SLO as “intact” and + SLO as “reseal”). Exogenously added FITC-BSA was observed only in the resealing sample. (**e**) TRPS analysis was performed for EVs derived from tunicamycin (Tm)-treated or Tm-untreated cells. After post-incubation for 120 min, resealed HeLa cells were incubated in a 5% CO_2_ incubator for 24 h at 37 °C in the presence or absence of 5 µg/mL of Tm, and then EVs from each sample were prepared and analyzed by TRPS analysis, as described in (**c**). The minimum number of events recorded was 792 particles/measurement. The mean RMS noise was 4.7 ± 1.3 pA. The membrane stretch range used was 47.5 nm, the voltage used was 0.50 V, and the current range used was 104.6–116.5 nA. (**f**) Fluorescence microscopy images of EVs endocytosed by intact HeLa cells. EVs prepared from intact or resealed HeLa cells, as described in (**b**), were stained with 2 µM PKH26 dye. Fluorescence-labeled EVs from intact or resealed HeLa cells were incubated with intact HeLa cells in a 5% CO_2_ incubator for 24 h at 37 °C. The cells were fixed and observed by confocal microscopy. Fluorescence-labeled EVs from resealed HeLa cells were endocytosed by HeLa cells like EVs from intact HeLa cells. White dotted lines outline the cell. Scale bar = 20 µm.

To reconstitute the EV secretion in resealed HeLa cells, we first evaluated whether we could accomplish EV formation in and their subsequent secretion from resealed HeLa cells. According to the resealing protocol described previously^24,25^, we prepared resealed HeLa cells in which the cytosol was replaced with that prepared from murine lymphoma L5178Y cells (protein concentration ~ 3 mg/mL), which has been successfully used for reconstitution of intracellular membrane dynamics, such as cell cycle-dependent organelle dynamics or the membrane traffic between organelles in cells (Fig. 1a)^21,22^. The resealing efficiency, which was determined by flow cytometric and morphometric analyses, was ~ 90% (Supplementary Fig. S1), as previously reported^24,25^.

SLO treatment for preparing semi-intact cells promotes ectocytosis and activates the production of plasma membrane-derived EVs, and Ca^2+^ influx during cell resealing promotes lysosomal exocytosis^23^. These stress responses potentially affect experimental results for elucidating the mechanisms underlying EV formation in resealed cells. To estimate these stress responses of resealed HeLa cells, we first examined the activation of stress-responsive kinases, such as p38 mitogen-activated protein kinase (p38 MAPK) and c-Jun N-terminal kinase (JNK). After resealing, the resealed HeLa cells were incubated in a 5% CO_2_ incubator for 0, 30, 60, and 120 min at 37 °C and then subjected to western blotting (WB) or immunofluorescence (IF) analysis using antibodies against p38 MAPK, JNK, and their phosphorylated forms. We observed little or no activation of JNK during resealing. In contrast, p38 MAPK was activated (phosphorylated) just after resealing but went back to the original state within ~ 30 min (Supplementary Fig. S2. Similarly, IF analysis showed that the fluorescence signal of phosphorylated p38 MAPK in the nucleus (activated) decreased to the original state within 60 min (Supplementary Fig. S3). These results indicated that at least 60 min of further incubation of resealed HeLa cells after resealing (called post-incubation) is likely enough to restore the possible effects of the stress-induced by cell resealing on EV formation in resealed HeLa cells.

Furthermore, we performed ultramicrostructural analysis of isolated EVs by negative-stained electron microscopy (EM). After 120 min post-incubation, we changed the medium of resealed HeLa cells with fresh medium containing EV-depleted serum and further incubated the cells for 48 h. Incubation for 48 h was used to obtain a sufficient amount of EVs from the medium of resealed HeLa cells for usual morphological or biochemical analysis. EVs in the cell medium were concentrated by differential ultracentrifugation (referred to as UC EVs). Negative-stained EM showed that the UC EV morphology had no distinct structural difference between intact (control) and resealed HeLa cells, and both were 100–200 nm or smaller in diameter (Fig. 1b). Next, we obtained UC EVs as described above and measured the particle size distribution of the EVs by tunable resistive pulse sensing (TRPS). The particle size for resealed cell-derived EVs was nearly 100 nm in diameter, which was indistinguishable from EVs derived from intact HeLa cells under the same conditions (Fig. 1c). The measured size of resealed cell-derived EVs was consistent with that previously reported but totally different from that of membrane-derived apoptotic bodies^3^.

### Biochemical characterization of EVs derived from resealed HeLa cells

Next, we compared protein compositions of UC EVs derived from resealed and intact HeLa cells by WB analysis. We prepared resealed HeLa cells to which cytosol containing fluorescein isothiocyanate-bovine serum albumin (FITC-BSA) (protein concentration 100 µg/mL) was introduced. After post-incubation, we discarded the medium and further incubated resealed HeLa cells with fresh medium containing EV-depleted serum in a 5% CO_2_ incubator for 48 h at 37 °C. Next, we collected the resealed HeLa cell and EV fraction samples, dissolved them in radioimmunoprecipitation assay (RIPA) buffer, and subjected them to WB analysis. As a control, intact HeLa cells were pre-incubated with FITC-BSA for 25 min, followed by incubation with medium for 120 min. After washing the cells, they were further incubated them with fresh medium containing EV-depleted serum for 48 h, and intact HeLa cell and EV fractions samples were prepared similar to resealed HeLa cells. As shown in Fig. 1d (right column, intact and reseal), we detected standard EV marker proteins, such as TSG101, CD63, Flotillin-1, and glyceraldehyde 3-phosphate dehydrogenase (GAPDH). Especially, we detected the FITC-BSA band only in resealed HeLa cells, indicating that FITC-BSA in exogenously added cytosol is, probably, diffusively and passively incorporated into EVs. In contrast, we detected calnexin, a non-EV marker, only in the cell sample but not in the EV fraction sample (Fig. 1d). We obtained similar results with regard to protein components of cell and EV fraction samples using rat hepatoma H4IIEC3 resealed cells (Supplementary Fig. S4). Importantly, we detected no SLO band in either the cell samples or the EV fraction, indicating that EV fractions prepared from the medium of resealed HeLa cells do not contain microvesicles derived from the plasma membrane as SLO-induced blebbing (Supplementary Fig. S5). Because we replaced the medium after 120 min of resealing, most SLOs on secreted microvesicles in the medium were expected to be removed. Furthermore, some SLOs may have been retained on the plasma membrane or endocytosed in resealed cells^26^; however, most were expected to undergo degradation within 48 h as no SLO band was detected in the cell samples.

EV secretion into the culture medium is upregulated by oxidative stress^27^ or endoplasmic reticulum (ER) stress^28^. The ER stress-induced increase in EV production was also found in tunicamycin (Tm)-treated resealed cells (Fig. 1e and Supplementary Fig. S6). In addition, when EVs concentrated using Total Exosomes Isolation reagent (Invitrogen kit) from cell culture (referred to as TEI EVs, the morphological and biochemical characterization of which is described later) from intact and resealed HeLa cells were stained with PKH26, a lipid membrane staining agent, and incubated with HeLa cells for 24 h, PKH26-labeled EVs from resealed HeLa cells were incorporated into HeLa cells similar to those from intact HeLa cells (Fig. 1f). PKH26 could aggregate and undergo endocytosis by cells^29^; however, we could not detect any dots of PKH26 in phosphate-buffered saline (PBS) without EVs. These results indicated that the secretory response and cellular uptake ability of EVs obtained from intact and resealed HeLa cells are comparable.

Considering this biochemical data of EVs from resealed HeLa cells, together with the morphological information (Fig. 1), we believe that EV formation successfully occurred in resealed HeLa cells using exogenously added cytosol. In addition, we successfully introduced FITC-BSA into EVs (Fig. 1d).

### Entry of exogenously added cytosolic components into EVs using cell resealing

**Figure 2:**
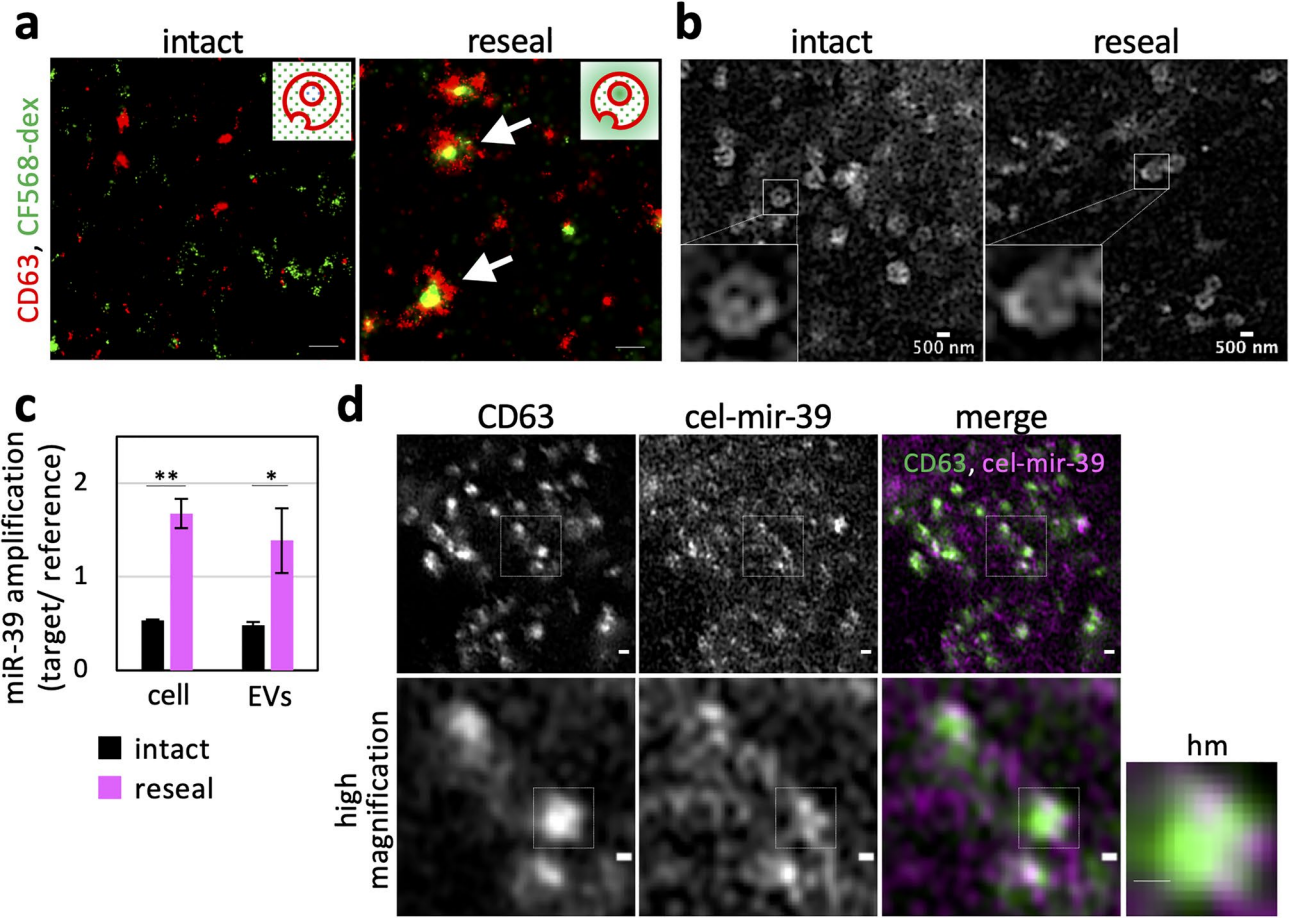
Encapsulation of exogenously added soluble cytosolic materials into ILVs and EVs in resealed HeLa cells. (**a**) Super-resolution images showing encapsulated 10 kDa CF568-labeled dextran (CF568-dex) in an intraluminal vesicle (ILV)-like structure. After introducing cytosol containing 100 µg/mL of CF568-dex, intact and resealed HeLa cells were post-incubated for 120 min and subjected to immunofluorescence (IF) analysis using anti-CD63 antibody (− SLO as “intact” and + SLO as “reseal”). The cells were observed by using an N-STORM super-resolution system. Green and red represent CF568 and CD63 signals, respectively. Arrows indicate CF568-dex incorporated in ILV-like structures. Inset: supposed models of ILV-like ultrastructure on background noise signals (dots). Scale bar = 200 nm. (**b**) Super-resolution images showing similarity of the ultrastructure of MVEs between intact and resealed HeLa cells. HeLa cells were incubated in DMEM without phenol red and observed using LSM980 with Airyscan2 at 37 °C (live-cell imaging). Inset: similar MVE-like ultrastructure observed in both intact and resealed HeLa cells. (**c**) Quantification of the amount of exogenously added synthesized miRNA in whole cells and EVs. The bar plot shows the relative amount of cellular- and EV-miRNA. After introducing cytosol containing a 1.0 nM synthesized cel-miR-39, intact and resealed HeLa cells were post-incubated for 120 min and further incubated in a 5% CO_2_ incubator for 48 h at 37 °C. Total cellular- and EV-RNA were extracted and miR-39-3p (guide strand) amount was measured by real-time PCR. Fluorescent signal values of the target (FAM) were normalized by those of references (ROX). Data represent results from three independent experiments (*n* = 3), expressed as the mean ± SD. *P < 0.05; **P < 0.01. (**d**) Super-resolution images showing Cy3-labeled miR-39-3p in the MVE-like structure. After introducing cytosol containing a 1.5 µM fluorescently labeled double-stranded miRNA mimic (Cy3 for cel-mir-39, guide strand; Cy5 for cel-mir-39*, passenger strand), resealed HeLa cells were post-incubated for 120 min and subjected to IF analysis using anti-CD63 antibody. The cells were observed using an LSM980 with Airyscan2. Arrows indicate miRNA accumulated in MVEs. The right panels are a magnified image of the white boxes in the left panel. hm: high magnification. Scale bar (upper images) = 500 nm. Scale bar (bottom images and cropped images) = 200 nm.

We examined whether ILVs in resealed HeLa cells are morphologically filled with the exogenously added soluble cytosolic materials. Resealed HeLa cells introduced with CF568-dextran (CF568-dex) were incubated in a 5% CO_2_ incubator for 120 min at 37 °C and subjected to IF analysis using anti-CD63 antibody. CD63 is expressed in various organelles (e.g., late endosomes, lysosomes, plasma membrane, other organelles), and it is particularly enriched in MVEs or ILVs^30^. As a control, we incubated intact HeLa cells with CF568-dex for 25 min in advance. ILVs immunostained with anti-CD63 antibodies in resealed HeLa cells were observed using super-resolution microscopy. The green CF568 signals appeared encapsulated in red dotted CD63 in resealed HeLa cells, while we observed little co-localization of CF568 spots with CD63 in intact HeLa cells (Fig. 2a). In our study, CF568-positive dots were distinguished from those labeled with endocytosed CF568 in intact HeLa cells topologically. In addition, the size of each green CF568 spot was ~ 200 nm in diameter, which is virtually identical to ILVs previously reported^31^. Super-resolution microscopy live imaging showed that the dynamics and morphology of EGFP-CD63-labeled MVEs in intact and resealed HeLa cells were almost indistinguishable (Fig. 2b and Supplementary Movies S1 and S2). These results indicated that the soluble cytoplasmic component in resealed HeLa cells can be encapsulated in exosomes in resealed HeLa cells and that the morphological features of the ILVs of MVEs in resealed HeLa cells are coincident with those previously reported^13,19,31^.

### Encapsulation of exogenously added miRNA into ILVs and exosomes in resealed HeLa cells

Subsequently, we examined the possible entry of exogenously added miRNA into ILVs and exosomes from cytosol diffusively in resealed HeLa cells. To test this biochemically, we used cel-miR-39 (QIAGEN) as a soluble and diffusive marker of miRNA. We prepared resealed HeLa cells using the cytosol containing cel-miR-39 and then obtained EVs from the medium of the resealed HeLa cells. It was reported that the combination of polymer-based precipitation method (Total Exosomes Isolation reagents reagent) and miRNeasy Mini kit (QIAGEN) had high extraction efficiency and provided highly pure small RNA^32^. We cannot eliminate the possibility that different subtypes of exosomes were obtained between the ultracentrifugation method and polymer-based precipitation method. However, we could not detect obvious differences in morphologies between UC EVs and TEI EVs via EM (Supplementary Fig. S7a). Moreover, we confirmed that TEI EVs also expressed known exosomal marker proteins, similar to UC EVs (Supplementary Fig. S7b). The concentrated EVs in PBS were subjected to RNase treatment to remove contaminated RNAs attached to the outside of EVs, and then RNA encapsulated in the EVs (EVs-RNA) was extracted. The same amount of EVs-RNA was used for reverse transcription and real-time PCR. We detected exogenously added cel-miR-39 in both resealed HeLa cells and EVs derived from the resealed HeLa cells (Fig. 2c, reseal), indicating that the incorporation of miRNA introduced into exosomes and their subsequent secretion to the extracellular region can occur in resealed HeLa cells. Concerning the origin of extracellular RNAs, no consensus has been reached^6^. Some researchers reported that most extracellular miRNAs originate from non-vesicular Ago2-associated miRNAs (mainly from dead cells and apoptotic bodies) that are resistant to RNase^33,34^. To reveal the origin of miRNA in TEI EVs from resealed cells, we examined the amount of exogenously added miR-39 as follows. First, we treated the TEI EV fraction with RNase alone and quantified the RNase-resistant miRNA by real-time PCR using the standard curve method (Supplementary Fig. S8). As a result, exogenously added miR-39 content was reduced but substantially detected even in the presence of RNase, indicating that this decreased amount of miRNA was likely to be extracellular miRNA free of proteins or EVs. Furthermore, we found that the aforementioned RNase-resistant miRNA was poorly degraded by proteinase K treatment in the presence of RNase, which can degrade protein-associated miRNA. We observed the complete disappearance of RNase-resistant miRNA only when the membrane components were disrupted by sodium dodecyl sulfate (SDS) in the presence of RNase. Together, these results suggested that RNase-resistant miRNA was encapsulated in TEI EVs.

To examine the subcellular localization of exogenously added miRNA, we focused on the possible interaction between Ago2 and miRNA, which are reported to form the RISC in the cytoplasm. First, we examined the subcellular localization of Ago2, CD63, and Dcp1 by IF using corresponding antibodies. Ago2 and Dcp1a are RNA-associated proteins, such as a marker of P-bodies that store RNA in the cytoplasm^35^. Ago2 was localized to either CD63 or Dcp1a extensively (CD63 and Ago2, localization efficiency; Pearson’s *r* = 0.474 ± 0.056; Dcp1a and Ago2, Pearson’s *r* = 0.416 ± 0.024), but there was little co-localization between CD63 and Dcp1a (Pearson’s *r* = 0.210 ± 0.032) (Supplementary Fig. S9a and S9b), indicating that Ago2 works as a recruiter of various types of RNAs for late endosomes or P-bodies separately, as previously reported^35^.

Ago2 unwinds short RNA duplexes, such as miRNA and short interfering RNA (siRNA), and stably associates with single-stranded RNA. This single-stranded RNA is called the guide strand (i.e., mature miRNA), while the opposite strand that is dissociated and undergoes degradation is called the passenger strand^6,7^. Next, we introduced a double-stranded miRNA mimic (cel-miR-39) for which the 5′-end of the guide strand was labeled with Cy3 and the 5′-end of the passenger strand with Cy5 into resealed HeLa cells, in which EGFP-CD63 or EGFP-Dcp1a was transiently expressed, and we investigated the co-localization of fluorescent miRNA and EGFP-CD63/EGFP-Dcp1a in fixed resealed HeLa cells 120 min after resealing using confocal microscopy. Both strands of the miRNA introduced in resealed HeLa cells appeared to accumulate with CD63-positive but not Dcp1a-positive structures (Supplementary Fig. S9c). Super-resolution microscopic analysis for close examination of the co-localization of CD63 with the Cy3-labeled guide strand in resealed HeLa cells by IF analysis using anti-CD63 antibody showed that the guide strand appeared to accumulate in the vicinity of CD63-positive structures, forming ILV-like structures (Fig. 2d). The CD63-positive structure was ~ 500 nm in diameter, and the accumulated miRNA was ~ 200 nm in diameter. These results indicated that, at least, the Cy3-labeled guide strand introduced into resealed HeLa cells is transported into CD63-positive MVEs via an innate cargo-sorting system.

### Time-dependent behavior of double-stranded miRNA introduced into resealed HeLa cells

**Figure 3:**
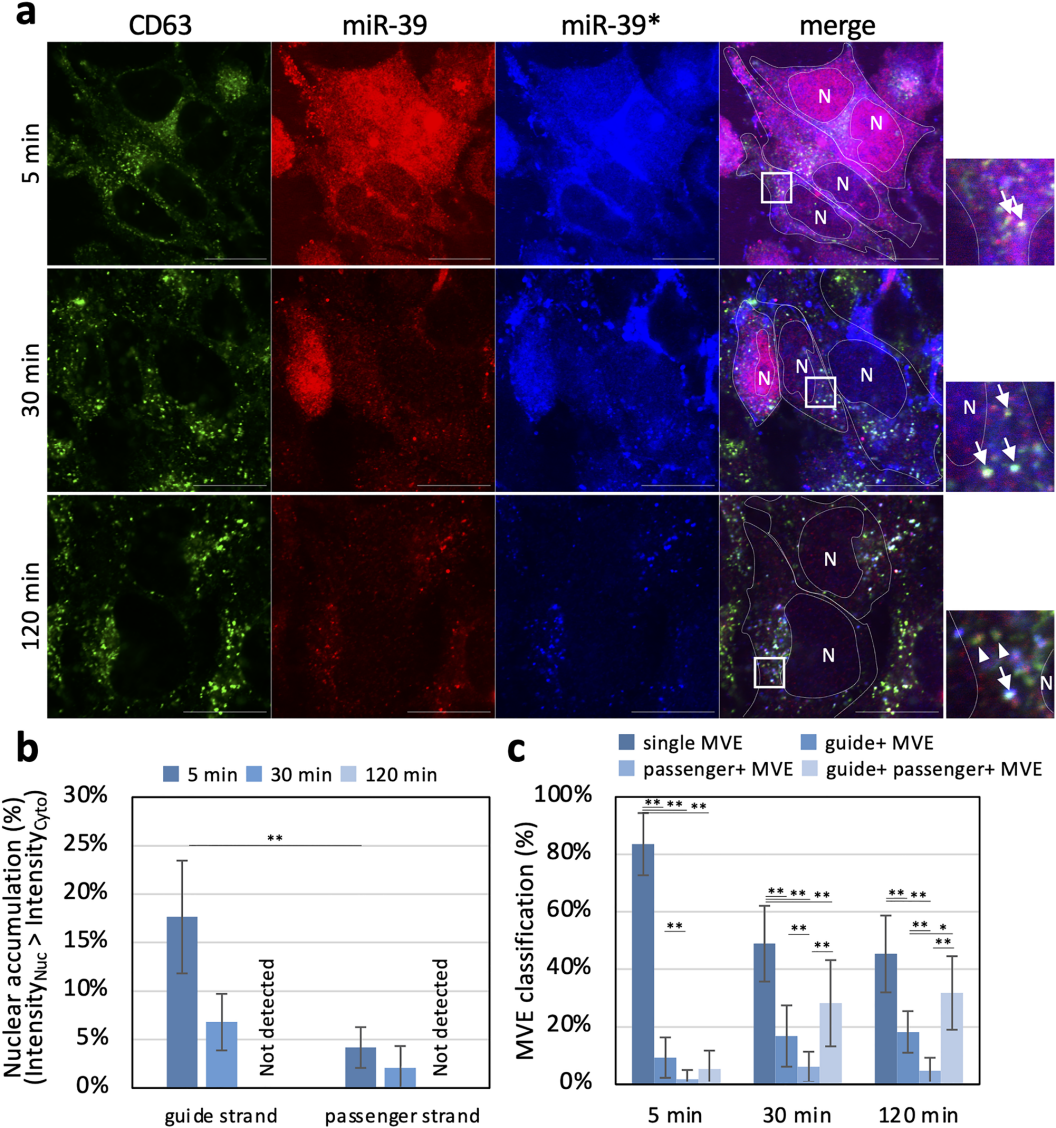
Time-dependent localization changes of exogenously added fluorescent-labeled strands of miRNA introduced into resealed HeLa cells. (**a**) Fluorescence microscopy images of the miRNA introduced in resealed HeLa cells. After introducing cytosol containing a 1.5 µM fluorescently labeled miRNA mimic, resealed HeLa cells were fixed at 5, 30, and 120 min after resealing and subjected to IF analysis using anti-CD63 antibody. Fluorescence signals of guide (miR-39) strands, passenger (miR-39*) strands, and CD63-positive MVEs were observed by confocal microscopy. Arrows indicate CD63-positive MVEs co-localized with both guide and passenger strands. Arrowheads indicate CD63-positive MVEs co-localized with the guide strand but not with the passenger strand. White dotted lines outline the cell and nucleus. Nuclear regions are indicated as “N” inside the dotted line. Scale bar = 20 µm. (**b**) Bar plots show the population ratio of cells in which miRNAs were accumulated in the nucleus. Data represent results from five frames (5 points and ~ 80 cells/sample; *n* = 5), expressed as the mean ± SD. *P < 0.05; **P < 0.01. (**c**) Bar plots show the population ratio of MVEs classified by the miRNA co-localization manner. For counting, we processed images using Laplacian of Gaussian (LoG) filtering to remove noise. Observed MVEs were selected at random (20 points/cell) and classified by co-localized miRNA bright spots. Data represent results from 14 cells (*n* = 14), expressed as the mean ± SD. *P < 0.05; **P < 0.01. single, no co-localized; guide + , co-localized with guide strand, passenger + MVE, co-localized with passenger strand.

Next, we investigated time-dependent changes of localization of the introduced fluorescently labeled miRNA in resealed HeLa cells. HeLa cells were introduced with fluorescently labeled cel-miR-39, and the resealed HeLa cells were incubated in a 5% CO_2_ incubator for 5, 30, and 120 min at 37 °C and then subjected to IF analysis using anti-CD63 antibody. After 5 min incubation, both guide (Fig. 3a, miR-39) and passenger (Fig. 3a, miR-39*) strands were distributed throughout the cells, but guide strands were more accumulated in the nucleus compared to passenger strands. Fluorescence spots of either the guide or the passenger strand or both of them began to emerge in the cytoplasm after 30 min incubation. After 120 min incubation, the guide strand’s fluorescence signal was eliminated from the nucleus (Fig. 3a,b), while fluorescence spots of both guide and passenger strands appeared in the cytoplasm (Fig. 3a,c). Quantitative image-based microscopic analysis showed that CD63-positive dots are co-localized mainly with fluorescence spots of the guide or guide/passenger strand of miRNA, not the passenger strand (Fig. 3c), which seemed reasonable because the passenger strand is easily degraded in the cytoplasm after separating from the guide strand^6,7,36^. As described earlier, < 120 min after resealing, resealed HeLa cells might be under stress induced by resealing, so we could not exclude the possibility that the dynamic behavior of fluorescently labeled miRNAs in 5 or 30 min incubation samples showed the behavior under stress. However, the transport of exogenously added miRNA to late endosomes (or CD63-positive MVEs) within 120 min almost entirely in resealed HeLa cells.

The miRNA was strongly co-localized with CD63 (MVEs) and Lamp2 (lysosomes) (Supplementary Fig. S10, arrow), but weakly co-localized with Rab7 (late endosomes) and Rab11 (recycling endosomes). A part of the passenger strand was co-localized with CD63 and Lamp2, together with the guide strand. However, the passenger strand was mainly co-localized with the organelle-nonspecific guide strand (Supplementary Fig. S10, arrowhead). These morphological and biochemical results indicated that we could reconstitute miRNA transport to ILVs and exosomes in resealed HeLa cells.

### Inhibition of protein functions by introduced antibody in resealed HeLa cells

We focused on Ago^8,9^ and YBX1^15,37,38^, which are RBPs that interact with a large family of proteins and RNA species, including miRNA, as representative cytosolic factors. We prepared the modified cytosol, which contained either anti-Ago antibody or anti-YBX1 antibody, and examined its effect on the inhibition of miRNA transport to MVEs or EVs in resealed HeLa cells by biochemical and morphological analyses. WB analysis using anti-pan-Ago, anti-Ago2 and, anti-YBX1 antibodies showed that YBX1 is present in HeLa cells and EVs, whereas pan-Ago and Ago2 were present only in HeLa cells but barely detected in EVs (Supplementary Fig. S11a). Immunoprecipitation and immunodepletion experiments for HeLa cell lysates and L5178Y cytosol, respectively. The intensity of the upper band (Ago; 90–100 kDa) in the antibody lane was clearly diminished. The lower band (Ago; nearly 70 kDa) was a cross-reacting band (Radixin)^39^ that was not diminished. This suggests that the anti-pan-Ago antibody recognized the full-length Ago in resealed HeLa cells. In the case of anti-YBX1 antibody, a decrease in YBX1 intensity was confirmed. These results showed that these antibodies significantly interacted with each antigen, even in resealed HeLa cells (Supplementary Fig. S11b). Next, to examine the resealing efficiency and retention rate of introduced immunoglobulin G (IgG)-type antibodies in resealed HeLa cells in which normal mouse IgG or normal rabbit IgG was introduced by resealing, the resealed HeLa cells were incubated in a 5% CO_2_ incubator for 12 h at 37 °C and then subjected to IF analysis using anti-mouse and anti-rabbit antibodies. We detected fluorescence signals of both antibodies in the cytoplasmic region but not in the nuclear region in almost all resealed HeLa cells (~ 90%), even after 12 h incubation (Supplementary Fig. S11c).

**Figure 4:**
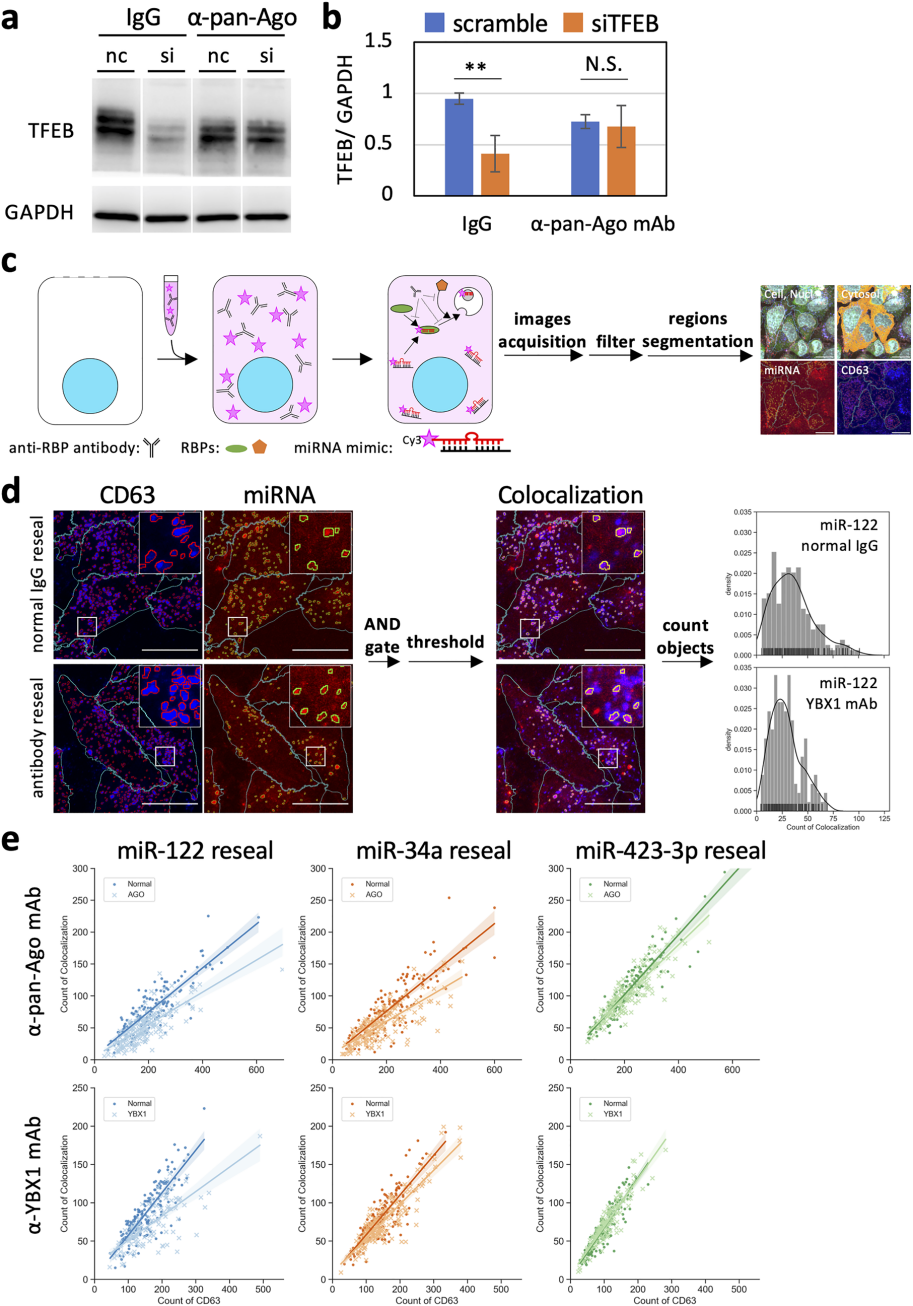
Quantitative image-based microscopic analysis of exogenously added fluorescent-labeled miRNAs loading to MVEs in resealed HeLa cells containing function-blocking antibodies. (**a**) Functional analysis of exogenously added antibody in resealed cells by WB analysis. After introducing cytosol containing 10 µg/mL of antibodies, HeLa cells were post-incubated for 45 min, transfected with the siRNA by lipofection, and further incubated in a 5% CO_2_ incubator for 18 h at 37 °C. Next, the cells were lysed and subjected to WB analysis using anti-TFEB and anti-GAPDH antibodies. Equivalent band intensities between scramble (nc) and siRNA (si) in resealed HeLa cells were observed ($\alpha$-pan-Ago). (**b**) Quantification of TFEB and GAPDH bands in (**a**). The bar plot shows the relative TFEB amount in resealed HeLa cells. The TFEB amounts were compared, normalized by GAPDH. Data represent results from three independent experiments (*n* = 3), expressed as the mean ± SD. **P < 0.01. (**c**) Computer algorithm-defined segmentation scheme. After introducing cytosol containing 1.5 µM fluorescently labeled miRNAs and 10 µg/mL of anti-Ago or anti-YBX1 antibodies, HeLa cells were post-incubated for 120 min and subjected to IF analysis using anti-Ago2 and anti-CD63 antibodies. Mouse anti-CD63 antibody was used to reseal HeLa cells containing rabbit anti-YBX1 antibody, and rabbit anti-CD63 antibody was used to reseal HeLa cells containing mouse anti-pan Ago antibody. We obtained ~ 100 images (9 points × 9 Z-stack images) per sample and performed maximum-intensity projection prior to image analysis. We detected the areas of nuclei, cells, miRNAs, and MVEs and performed LoG filtering, threshold setting, and AND-gated image calculation for each image. Next, we counted the number of objects automatically at the single-cell level. Scale bar in the right panel = 20 µm. (**d**) Detection scheme of CD63-positive MVEs, miRNA dots, and their co-localization. Images were filtered using the AND gate, and co-localized objects were enumerated automatically. The histogram shows the results of resealed HeLa cells containing miR-122 and anti-YBX1 antibody [lower-middle panel in (**e**)]. Scale bar = 20 µm. (**e**) Scatter plots with linear regression models for quantitative results obtained, as described in (**c**, **d**). Scatter plots with linear regression models show the number of CD63-positive MVEs and co-localized objects. The 95% confidence intervals were drawn using translucent bands around regression lines.

In addition, to confirm the inhibitory function of exogenously added antibody in cells, we examined the effect of the introduced anti-pan-Ago antibody on the siRNA-mediated knockdown efficiency. Resealed HeLa cells were incubated in a 5% CO_2_ incubator for 45 min at 37 °C and then transfected with the siRNA for silencing of transcription factor EB (*TFEB*), a master transcriptional regulator of lysosomal biogenesis and autophagy, as a representative sample. After incubation for 18 h, the cells were lysed and subjected to WB analysis. The knockdown efficiency of control resealed HeLa cells, in which normal IgG was introduced, was ~ 50%, while that of sample resealed HeLa cells, in which anti-pan-Ago antibody was introduced, was nearly 0, same as that of the scrambled control (Fig. 4a,b). These results indicated that the anti-Ago and anti-YBX1 antibodies introduced in resealed HeLa cells could recognize and interact with antigen proteins, inhibiting their functions, specifically in resealed HeLa cells.

### Quantitative analysis of subcellular localization of miR-122, miR-34a, and miR-423-3p in resealed HeLa cells and the effect of antibodies against Ago and YBX1

After achieving the transport and encapsulation of exogenously added miRNA into exosomes and EV secretion in resealed HeLa cells, we examined the effect of anti-Ago and anti-YBX1 antibodies on the transport of miRNA mimics to CD63-positive MVEs. We selected these miRNAs (miR-122^40–43^, miR-34a^40,44,45^, and miR-423-3p^40,46,47^) because they are expressed in HeLa cells^40,41,44,46^. Furthermore, they interact with Ago proteins^16,42,43,45,47^, and studies have investigated their sorting mechanism^16,18,46^. Three types of miRNAs, in which guide miRNA was conjugated with Cy3, were introduced into HeLa cells in the presence of control IgG, anti-Ago antibody, or anti-YBX1 antibody by cell resealing. After 120 min, the cells were fixed and stained with anti-CD63 antibody. Fluorescent spots of exogenously added miRNA were heterogeneously distributed throughout the cytoplasm and we saw many fluorescent spots that were positive only with CD63 or with both CD63 and miRNA, which made it difficult to estimate the co-localization rate between miRNA and CD63 visually. Therefore, we performed automatically quantitative image-based microscopic analysis. For single-cell analysis, HeLa cells were stained with Hoechst 33,342 (for nucleus staining) and anti-Ago2 antibody (for cytosol staining) for segmentation of each cell (Fig. 4c). Anti-Ago2 antibody was used as a marker of the cytoplasm because Ago2 was evenly distributed throughout the cytoplasm. To quantify the co-localization of miRNA and CD63-positive MVEs in each cell, miRNA- or CD63-positive dotted structures were segmented (Fig. 4d). To prevent secondary antibodies from recognizing the antibodies incorporated into resealed HeLa cells, the host species of the primary antibodies were different from the antibodies in resealed HeLa cells (Supplementary Fig. S12). The mean co-localization rates of miR-122, miR-34a, and miR-423-3p, with CD63-positive MVEs, were 48.2% ± 14.6%, 48.0% ± 14.4%, and 60.8% ± 12.7%, respectively, in the presence of control IgG, indicating that miR-423-3p might be more actively transferred to CD63-positive MVEs compared to other miRNAs. The co-localization of miR-122 and miR-34a with CD63 appeared slightly repressed by the addition of anti-Ago antibody (Fig. 4e, α-pan-Ago antibody row). Interestingly, anti-YBX1 antibody significantly decreased the co-localization of miR-122 with CD63-positive MVEs (Fig. 4e, α-YBX1 antibody row). In contrast, the addition of these antibodies did not affect the subcellular localization of miR-423-3p (Fig. 4e, miR-423-3p reseal column). Statistical analysis revealed that the co-localization rates of miR-122 and miR-34a with CD63 were decreased by the addition of anti-Ago antibody, and the co-localization of miR-122 with CD63 was also decreased by the addition of anti-YBX1 antibody (Supplementary Fig. S13). Collectively, anti-Ago and anti-YBX1 antibodies displayed different effects on the localization of three types of miRNA to CD63-positive late endosomes; especially, the localization of miR-122 to CD63 depended on YBX1 in HeLa cells.

### Type-dependent encapsulation of miRNA into secreted EVs in resealed HeLa cells

**Figure 5:**
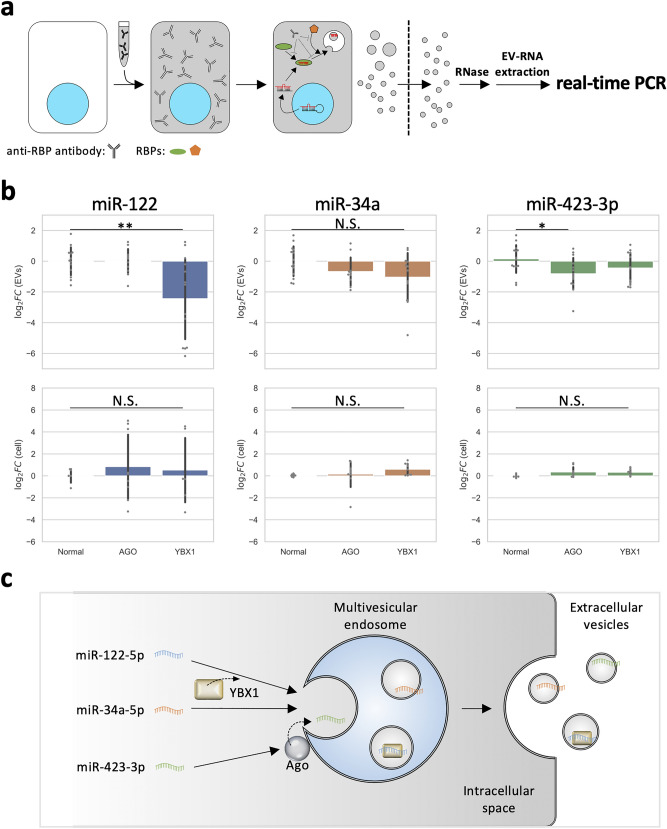
Quantitative analysis of miRNAs in isolated EVs derived from resealed HeLa cells, in which function-blocking antibodies against RBPs were included. (**a**) Experimental scheme examining the effect of function-blocking antibodies against RNA-binding proteins (RBPs) on loading of various endogenous miRNAs in resealed HeLa cells. After introducing cytosol containing 10 µg/mL of anti-Ago or anti-YBX1 antibodies, HeLa cells were incubated in a 5% CO_2_ incubator for 12 h at 37 °C. Total cellular- and EV-RNA were obtained, as described in Fig. 2c, and the miR-122, miR-34a, and miR-423-3p amounts were measured by real-time PCR. (**b**) Quantification of the amount of endogenously expressed miRNA in whole cells and EVs. Bar plots show the relative cellular- and EV-miRNA amounts. Log2 fold-change (FC) values of each miRNA were calculated using the $\mathrm{\Delta \Delta }$ Ct method, in which Ct was normalized to 18S rRNA and $\Delta$ Ct to the control sample (normal). When log2 FC values were greater than two times standard deviation (SD), we assumed them to be outliers and excluded them. We selected TaqMan assays, which have the same sequences between humans and animals. Therefore, TaqMan assays are presumed to detect them. EVs; miR-122, *n* = 10–13; miR-34a, *n* = 10–14; miR-423-3p, *n* = 12–14. Cell; miR-122, *n* = 5–9; miR-34a, *n* = 9; miR-423-3p, *n* = 9–10. *P < 0.05; **P < 0.01. (**c**) Possible model of loading mechanism of each miRNA to EVs. Considering all results concerning different efficiencies for recruiting to MVEs among miRNAs (Fig. 4d) for miRNA loading to exosomes in (**b**), we schematically proposed a possible model for loading of miRNA to exosomes. Both the loading and recruiting mechanisms would depend on the miRNA species.

Next, we evaluated the encapsulation of endogenous miR-122, miR-34a, and miR-423-3p in secreted EVs. We prepared resealed HeLa cells using cytosol containing control IgG, anti-Ago antibody, or anti-YBX1 antibody and obtained EVs from the medium containing the EV-depleted serum of the resealed HeLa cells 12 h after resealing. Then the same amount of RNA in secreted EVs and the remained resealed HeLa cells were transcribed, and the amount of endogenous miRNA was measured by real-time PCR (Fig. 5a). Anti-Ago or anti-YBX1 antibody decreased the amount of miR-122 and miR-423-3p in secreted EVs, respectively (Fig. 5b). Although miR-122, miR-34a, and miR-423-3p were expressed at lower levels than miRNAs, such as miR-21 and let-7a in HeLa cells^40^, we could quantify with sufficient sensitivity the amounts of these miRNAs by real-time PCR in HeLa cells and HeLa cell-derived EVs. Since the addition of these antibodies did not affect the amount of cellular miRNA, miRNA degradation did not occur in their presence. In addition, to estimate the effect of resealing stress on miRNA quantification, endogenous miRNAs in cells and EVs were prepared after post-incubation of resealed HeLa cells, and miR-122 and miR-34a amounts were measured by real-time PCR. There was little difference in Ct values between the intact and resealed conditions (Supplementary Fig. S14b).

Furthermore, we examined the amount of endogenously expressed miRNA (miR-122, miR-34a, and miR-423-3p) following proteinase K or SDS treatment in the presence of RNase, as described previously. We found that endogenously expressed miRNAs content was reduced but detectable, and these RNase-resistant miRNAs were poorly degraded by proteinase K but completely disrupted by SDS in the presence of RNase, as noted for exogenously added miR-39, indicating that the detected extracellular miRNAs mainly originated from EVs.

The decrease in the amount of EV-loaded miR-122 by anti-YBX1 antibody seemed plausible because the antibody inhibited miR-122 transport to CD63-positive MVEs (Fig. 4e). In contrast, fluorescently labeled miR-423-3p exhibited mostly normal accumulation with CD63-positive MVEs in the presence of anti-Ago antibody, while anti-Ago antibody subtly decreased the amount of miR-423-3p in EVs. These results indicated that miR-423-3p can be recruited to CD63-positive MVEs but cannot be loaded to ILVs as cargo. The loading process requires RBPs, that of miR-122 depends on YBX1, and that of miR-423-3p depends on Ago. Overall, the transport of miRNAs to late endosomes and their encapsulation to EVs followed a distinct pathway regulated by RBPs, such as YBX1 and Ago, depending on miRNA types (Fig. 5c).

## Discussion

One important aim of this study was to reconstruct EV formation in the cell-resealing system using exogenously added cytosol. Via morphological and biochemical characterization of EVs derived from resealed cells, the system will be useful for not only elucidating the regulatory mechanisms underlying EV formation in living cells but also introducing various soluble biomaterials, such as proteins, miRNAs, and membrane-impermeable biopharmaceuticals, into EVs. EV formation pathways depend on novel RBPs, such as Ago and YBX1.

Exogenously added materials, such as FITC-BSA or fluorescently labeled miRNA, can be incorporated into EVs by cell resealing, and the incorporation appears to be via ILVs, which are concave membrane structures in CD63-positive MVEs, in resealed HeLa cells. The miRNA in resealed cells can be actively recruited to MVEs or ILVs, which depends on the miRNA type. In contrast, FITC-dex or FITC-BSA diffuses throughout the cells or only the cytoplasm, respectively. Exclusion of FITC-BSA (66 kDa) from the nucleus is due to the size-dependent barrier of nuclear pores, which prohibits free diffusion of > 40 kDa proteins across the nuclear membrane. The even distribution of FITC-dex and FITC-BSA throughout the cytoplasm indicates the possibility of passive diffusion of these molecules into EVs. However, endothelial cells secrete BSA-enriched EVs via transcytosis, which might regulate the metabolic state of adipose tissue^48^. Therefore, we cannot exclude the possibility of selective transport of FITC-BSA to EVs.

Previous studies reported that the majority of extracellular miRNA could be Ago2-associated instead of EV-associated^33,34^. We found that exogenously added miR-39 and endogenously expressed miRNAs levels were reduced but detectable in the presence of RNase, and they disappeared completely only when the membrane components were disrupted by SDS. In addition, there was little Ago2 in the UC EV or TEI EV fraction. Together, these results suggested that at least the RISC-derived miRNAs formed around Ago were not the main origin of extracellular miRNAs in TEI EVs under our experimental conditions, and more importantly, the RNase-resistant miRNAs were certainly encapsulated in TEI EVs. In the present study, we usually utilized the RNase-resistant miRNAs as the EV-loaded miRNA in the resealed cell EVs. These results indicate that EVs from resealed cells might become a powerful tool for drug delivery when we understand the mechanisms by which exogenously added molecules of interest could be loaded to EVs by cell resealing.

Cell resealing coupled with quantitative image-based microscopic analysis is suitable for investigating the biochemical requirements for miRNA-loaded EV formation in cells and for determining the mechanism underlying this formation, because the analytical system allows us to dissect concerted biological reactions into several elementary ones, biochemically and morphologically. One of the advantages of the cell-resealing system is to investigate the biochemical requirements for reconstituted biological reactions by introducing modified cytosol. For example, the addition of an antibody, which can inhibit protein functions in cells, into cytosol is the simplest way to modify cytosol.

By introducing cytosol containing fluorescently labeled miRNA into resealed cells, we can detect the subcellular localization, approximate amount, and aggregation states of miRNA in MVEs or ILVs in cells. Time-dependent changes of subcellular localization or the states of introduced fluorescently labeled miRNA can also be analyzed using IF analysis. In cells fixed 5 min after resealing, some of the cytoplasmic miRNA spots overlap with MVEs, indicating that the translocation of some type of miRNA to MVEs is rapid. Especially, the whole miRNA signal in cells weakens within 30–120 min, probably because of its degradation^49^, whereas fluorescent spots of miRNA in the cytoplasm become clearer. IF analysis shows that the bright spots of miRNA are mainly co-localized with CD63-positive MVEs but not with Dcp1a-positive P-bodies. We cannot exclude the possibility of some interference by endogenous miRNA; however, we believe that the influence of endogenous miRNAs on the interaction between Ago or YBX1 and miRNA mimics is likely small because three miRNAs are endogenously expressed at low levels in HeLa cells, as described previously^40^. Moreover, introducing excess amounts of miRNA mimic could cause a competitive inhibition of RISC formation^36^. It has been reported that the absolute amounts of miRNAs are estimated to range 1 × 10^2^–1 × 10^5^ copies per cell (cpc)^50,51^. In our experiment, 1.5 µM fluorescently labeled miRNA mimic was introduced by resealing, and its levels were estimated to be nearly 2.4 × 10^6^ cpc, considering that the HeLa cell volume is 2.6 × 10^3^ µm^3^, as reported previously^52^. Therefore, we assumed that ≥ 10 times endogenous miRNA copies would have been introduced. Therefore, we speculate that the number of miRNAs introduced was sufficient to form RISC in the resealed cells.

In contrast, miRNA introduced by microinjection accumulates partially in P-bodies and other unknown bright spots in the cytoplasm^36,53^. Therefore, the mode of extranuclear efflux or cytoplasmic accumulation of the miRNA introduced depends on the miRNA species, their modifications (e.g., single- or double-stranded), or the methods of their introduction into cells (e.g., microinjection^36^, cationic polymer-based transfection^54,55^, gymnosis^56^, reporter expression system^57,58^, or cell resealing). In addition, cel-miR-39, which is a synthetic miRNA mimic, presumably does not have a target sequence in the human cells. This might result in no co-localization of fluorescently labeled miR-39 with P-bodies.

We suppose that fluorescently labeled miRNA may be incorporated a lot in cells by our cell-resealing system, forming brighter fluorescence spots of miRNA. In addition, miRNA, which is unprotected by RBPs, can be degraded by exoribonucleases such as 3′–5′ exoribonuclease 1 (Eri1) and small RNA-degrading nuclease (SDN) families in the cytoplasm^49^. Therefore, the signal of unprotected miRNAs diffused in the cytoplasm could be easily lost, enhancing miRNAs isolated from the cytoplasm by membranes or those protected by proteins. Overall, we believe that cytoplasmic miRNA in resealed cells rapidly accumulates to CD63-positive MVEs.

IF analysis using fluorescently labeled miRNA shows that the Cy3-labeled guide strand introduced mainly accumulates to CD63-positive MVEs in resealed cells. Double-stranded miRNA mimics might exist, which accumulate to CD63-positive MVEs or nonmembranous structures in resealed cells. However, chemical modifications, such as Cy3- or Cy5-labeling to miRNAs, have no effects on the intracellular behavior of modified miRNAs^35,36,56^. Therefore, although the passenger strand is degraded selectively in the cytoplasm^6^, exosomes containing both guide and passenger strands could exist. This idea is supported by recent reports that analyzed numerous passenger-strand miRNAs in exosomes of colon cancer cells^59,60^. Several questions, such as whether guide and passenger strands would remain annealed in exosomes, need to be clarified in the future.

To examine the involvement of the protein in each elementary process in EV formation, including specific miRNA in cells, our reconstitution system of miRNA transport to MVEs and EV secretion will allow easy and direct comparison of the assay of the direct protein function in processes with RNAi-/CRISPRi-mediated knockdown and CRISPR-mediated knockout methods. For the latter, complete depletion of the protein of interest usually takes a few days at least and disturbs the endosomal trafficking pathway because of transfection reagents. The direct effect of protein depletion leads to subsequent perturbation in various cellular processes, such as transcription, translation, and signal transduction. Therefore, the phenomenon observed in the target protein-depleted cells shows both direct and indirect involvement of the protein. Compared to these techniques, our reconstitution system allows the examination of the direct protein function in reconstituted processes.

In our reconstitution system, anti-YBX1 antibody represses the transport of miR-122 to MVEs and also decreases the amount of miR-122 in secreted EVs. There are several ways to inhibit miR-122-specific recruitment to MVEs by anti-YBX1 antibody: direct perturbation of YBX1–miRNA binding, functional abnormality through conformational changes by anti-YBX1 antibody binding, and perturbed interaction between YBX1 and other proteins. The anti-YBX1 antibody we used (clone EP2708Y) recognizes the unstructured C-terminal domain (CTD) of human YBX1 but not the RNA-interacting domain (cold-shock domain [CSD]) in the N-terminus, indicating that perturbation of the sequence-specific YBX1–miR-122 interaction through the CSD in the N-terminus is not the major reason for this inhibition and also indicating the novel function of the CTD as the exosomal miRNA sorting. In addition, the amount of miR-423-3p in secreted EVs significantly decreases by the addition of anti-pan-Ago antibody, while the localization of miR-423-3p to CD63-positive MVEs is almost normal. These findings indicate that a loading process of miRNA to EVs, whose molecular mechanism is distinct from that for recruiting miRNAs to MVEs, exists. Therefore, the novel role of Ago in miRNA type-dependent loading can be identified by dissecting miRNA encapsulation to EVs using our reconstitution system.

However, further studies are necessary to reveal the detailed mechanism of the observed differences between the behaviors of these miRNAs. It is known that Ago recognizes miRNAs through its conserved PAZ domain independently of miRNA sequences^8,9^, suggesting that the three miRNAs interact in the same manner; however, different specificities and selectivities, depending on the combination of cell type and miRNA species, have been reported. miR-122 interacts with and is sorted to exosomes with the La protein more efficiently than with Ago2 in MDA-MB-231 cells^18^. miR-34a increases Ago2 specificity via phosphorylation at the 5′-end in response to DNA damage in A549 cells^45^. The efficiency of miR-423-3p binding to Ago2 is increased by neuronal apoptosis in the hippocampus^47^. Furthermore, the requisite factors for miRNA encapsulation to EVs differ depending on miRNA types, cell types, and cellular conditions, probably because of the difference in the selectivity and affinity of each miRNA to respective RBPs or interactors, such as endogenously expressed RBPs^10^.

Our technique is widely applicable because most mammalian cells expressed cholesterol on their plasma membrane, which is essential for SLO binding and/or SLO-mediated pore formation. Using our resealing cell technique, we can introduce various types of cargoes, such as siRNA, membrane-impermeable proteins, peptides, metabolites, and functional aptamers, into a variety of mammalian cells. Further experimentation, such as high-efficient EV isolation methods^61^ or stoichiometric analysis^62–64^ coupled with our system, may be useful for future studies. We believe that our reconstitution system using resealed cells would be beneficial for determining which proteins are involved in the elementary processes of cargo encapsulation to EVs under specific cellular conditions.

One interesting application of our cell-resealing system is to analyze disease-specific EVs derived from disease model cells where pathological cytosol is introduced into cells by cell resealing. For example, a diabetic hepatocyte model was prepared by introducing cytosol prepared from the liver of leptin receptor-deficient type2 diabetic db/db mice model. The hepatocytes reproduced the typical pathogenic phenotype, insulin resistance, that is, insulin treatment did not repress glucogenesis-related gene expression^25^ and the abnormal enhancement of epidermal growth factor receptor (EGFR) internalization^24^, which was also reported in the primary hepatocytes of diabetic model rats^65,66^. Therefore, comparing the components or physicochemical properties of EVs derived from disease model cells to healthy model cells would help identify various disease-specific markers in EVs with their cell-type origins. This would be unique for our assays since body fluids include EVs from various tissues, making it challenging to determine the origins of EVs and the tissues in which these disease markers represent abnormalities.

In conclusion, our cell-resealing system for creating EVs from resealed cells is a novel and powerful tool for EV basic and applied studies. EV formation in resealed cells can be used not only to create a reconstitution system to give mechanistic insight into EV encapsulation but also for applications such as loading molecules of interest into EVs and identifying disease-specific EV markers.

## Methods

### Reagents

Adenosine triphosphate (ATP), creatine phosphate, creatine kinase, and guanosine triphosphate (GTP) were purchased from Sigma-Aldrich (St. Louis, MO, USA). Bovine serum albumin (BSA) conjugated with fluorescein, and 10 kDa dextran conjugated with fluorescein were purchased from Invitrogen (Carlsbad, CA, USA), while 10 kDa dextran conjugated with CF568 was purchased from Biotium (Fremont, CA, USA). A 0.22-µm Millex-GV Filter (#SLGV004SL) was purchased from Millipore (Burlington, MA, USA). Hoechst 33,342 solution was purchased from Dojindo (Kumamoto, Japan), CellMask Deep Red was obtained from Thermo Fisher Scientific (Waltham, MA, USA), and propidium iodide (PI) was procured from Molecular Probes (Eugene, OR, USA). miRNA spike-in control (cel-miR-39) from QIAGEN (Hilden, Germany), miRNA mimic conjugated with Cy3 or Cy5 (hsa-miR-34a, MI0000268; hsa-miR-122, MI0000442; hsa-miR-423, MI0001445; cel-miR-39; MI0000010) from Integrated DNA Technologies (Coralville, IA, USA), and scramble miRNA mimic conjugated with fluorescein (SMC-4001) from Bioneer (Daejeon, Republic of Korea). siRNA duplex for *TFEB* knockdown from Dharmacon (D-009798–03-0005; Lafayette, CO, USA), and scrambled siRNA from Ambion (Austin, TX, USA). Antibodies and primers were purchased as described in Supplementary Table S1 and S2.

### Cell culture

We cultured HeLa cells (previously existing collection in the Murata Laboratory at the University of Tokyo) in Dulbecco’s modified Eagle’s medium (DMEM; Nissui Pharmaceutical, Tokyo, Japan) containing 10% fetal calf serum (FCS; Sigma-Aldrich) and 1% penicillin/streptomycin (GIBCO, Co., Dublin, Ireland). We also cultured rat hepatoma-derived H4IIEC3 cells (ATCC) in DMEM (Nissui Pharmaceutical) containing 20% horse serum (ATCC), 5% FCS, and penicillin/streptomycin (GIBCO). Exosome-depleted fetal bovine serum (FBS) (Exo-FBSHI; System Biosciences, Palo Alto, CA, USA) was used for EV isolation. To generate stress-induced EVs, we cultured HeLa cells with or without 5 µg/mL of Tm (Wako, Osaka, Japan) for 24 h. Finally, we counted the number of living HeLa cells using Cell Counting Kit-8 (CCK-8; Dojindo).

### Cytosol preparation

We prepared cytosol from murine lymphoma L5178Y cells, as described previously^24^*.*

### Resealed HeLa cell preparation

We performed the resealing protocol, as described previously^24,25^. Briefly, HeLa or H4IIEC3 cells were washed twice with PBS and then incubated for 5 or 10 min with 100 or 200 ng/mL of SLO (Bioacademia, Osaka, Japan) on ice, respectively. HeLa or H4IIEC3 cells were again washed thrice with PBS, incubated with transport buffer (TB: 25 mM 4-(2-hydroxyethyl)-1-piperazineethanesulfonic acid [HEPES]-KOH [pH 7.4], 115 mM potassium acetate, 2.5 mM MgCl_2_, and 2 mM ethylene glycol-bis(β-aminoethyl ether)-*N*,*N*,*N*,*N*-tetraacetic acid [EGTA]) for 10 or 5 min at 37 °C, respectively, and again washed with TB without EGTA. If required, we supplemented TB with 300 µg/mL of PI for incubation. The obtained cells were called semi-intact cells. Next, HeLa or H4IIEC3 cells were incubated with 3.0 mg/mL of L5178Y cytosol containing an ATP-regenerating system (1 mM ATP, 50 µM creatine kinase, and 2.62 mg/mL of creatine phosphate), 1 mg/mL of glucose, and 1 mM GTP for 20 or 30 min at 37 °C, respectively. If required, we supplemented cytosol with fluorescently labeled beacons (100 µg/mL of dextran, 100 µg/mL of BSA, 1.5 µM miRNA mimic, or 1.0 µM scramble miRNA mimic), 1.0 nM spike-in control (cel-miR-39; QIAGEN), or 10 µg/mL of antibodies. Next, we added 1 mM CaCl_2_ at a final concentration and incubated the cells for another 5 min at 37 °C. The cells were then washed thrice with PBS and were further incubated with prewarmed medium in a 5% CO_2_ incubator at 37 °C for the time indicated in the text.

### Preparation of EVs and EV-RNA

We prepared EVs from culture media of 3 × 10^6^ to 4 × 10^7^ HeLa or H4IIEC3 cells by differential centrifugation, as described previously^28,67–69^ with some modifications. Briefly, we collected culture media and centrifuged them at 2000 × *g* for 10 min at 4 °C. Next, the supernatant was passed through 0.22 µm filters to remove cell debris and large vesicles, such as microvesicles and apoptotic bodies.

We selected the following procedures optimally with each analysis. We precipitated UC EVs using ultracentrifugation with a P40ST rotor (Eppendorf Himac Technologies, Ibaraki, Japan) at 160,000 × *g* for 70 min at 4 °C. The EVs were suspended with 100 µL of PBS or RIPA buffer. We precipitated TEI EVs using Total Exosome Isolation Reagent from cell culture media (Invitrogen). Next, the EVs were suspended with PBS and incubated with 0.5 mg/mL RNase A (Millipore) for 30 min at 37 °C. Then, we extracted total RNA using the miRNeasy Mini Kit (QIAGEN) and stored it at − 80 °C. If required, EVs were stored at 4 °C and used within 1 week.

### Negative-stained electron microscopy

EVs were absorbed into carbon-coated nickel grids (400 mesh) or formvar film-coated copper grids and stained with 2% phosphotungstic acid solution (pH 7.0) for 10–60 s. The grids were observed under a JEM-1400 Plus transmission electron microscope (JEOL, Tokyo, Japan) at an acceleration voltage of 100 kV. Digital images (3296 × 2472 pixels) were captured with an EM-14830RUBY2 charged-coupled device camera (JEOL).

### Tunable resistive pulse sensing

We used the TRPS qNano IZON system (Izon, Christchurch, New Zealand) to measure the concentration and size distribution of EVs in PBS according to a previous study^70^ and the manufacturer’s instructions. The minimum number of events recorded was ≥ 100 particles/measurements. The maximum root mean square (RMS) noise was ≤ 10 pA. The current range was 80–120 nA. UC EVs obtained from theƒ same number of living cells were analyzed using NP200 nanopore and CPC100B calibration beads (110 nm). Data analysis was performed using qNano IZON software (v3.3.2, Izon).

### Cellular uptake

We suspended TEI EVs with PBS and reconcentrated them by ultrafiltration using 100 kDa MWCO Amicon Ultra-0.5 (Millipore). Next, EVs were stained with 2 µM PKH26 (Sigma-Aldrich) for 5 min at room temperature. After washing thrice with PBS, 1 µg/mL of the labeled EVs were added to ~ 70% confluent HeLa cells grown in 96-well plates. These HeLa cells were further incubated in a 5% CO_2_ incubator for 24 h at 37 °C and then fixed with 3.6% paraformaldehyde (PFA) for 20 min at room temperature. Finally, after washing thrice with PBS, the stained samples were examined under an A1 + confocal laser microscope (Nikon, Tokyo, Japan).

### Western blotting assay

We lysed HeLa cells or EVs using ice-cold RIPA buffer (50 mM Tris [pH 7.4], 150 mM NaCl, 1% NP-40, 0.25% sodium deoxycholate, 1 mM ethylenediaminetetraacetic acid [EDTA]) containing the cOmplete EDTA-free protease inhibitor cocktail and PhosSTOP (Roche, Baden, Switzerland). After homogenization, we measured the protein concentration using a BCA protein assay kit (Thermo Fisher Scientific). Next, we boiled the lysates in Laemmli SDS sample buffer for 5 min, subjected them to 5%–20% sodium dodecyl sulfate–polyacrylamide gel electrophoresis (SDS-PAGE) (1 µg EVs/lanes at the final concentration), and transferred the SDS-PAGE gels to a methanol-presoaked polyvinylidene difluoride (PVDF) membrane (Millipore). We blocked the PVDF membrane with 5% BSA and 0.1% Tris-buffered saline (TBS) Tween-20 for 1 h at room temperature, probed it with primary antibodies overnight at 4 °C, and then further incubated it with HRP-conjugated secondary antibodies for 1 h at room temperature. These antibodies were diluted with the Can Get Signal Immunostain Immunoreaction Enhancer Solution (Toyobo, Kagoshima, Japan). Finally, we visualized and analyzed immune complexes of the same amounts of protein using the ECL Western Blotting Detection Kit (GE Healthcare, Chicago, IL, USA) and the LAS-4000 mini imaging system (FUJIFILM, Tokyo, Japan). Antibody information is available in Supplementary Table S1. Full-length gel images are available at the end of Supplementary Information.

### Real-time PCR

We lysed HeLa cells or TEI EVs and purified total RNA using the miRNeasy Mini Kit (QIAGEN). The same amount of total RNA from each sample was transcribed (5–10 ng RNA/samples), as described next. MiRNAs were transcribed using the TaqMan Advanced miRNA cDNA Synthesis Kit (Thermo Fisher Scientific). We also transcribed the 18S rRNA using the ReverTra Ace qPCR RT Kit (Toyobo) for an internal control^71^. Next, we performed real-time PCR using the Fast SYBR Green Master Mix (Applied Biosystems, Foster City, CA, USA) for 18S rRNA and Stem-loop TaqMan Advanced MicroRNA assays (Thermo Fisher Scientific) for miRNA. Finally, we measured the amount of RNA using the StepOnePlus Real-Time PCR System (Applied Biosystems). Primer information is available in Supplementary Table S2.

### Immunofluorescence confocal microscopy

First, we grew HeLa cells on glass-bottomed dishes (#3911–035; Iwaki, Chiba, Japan), cover glasses (Matsunami, Osaka, Japan), or a glass-bottomed 8-well cell chamber (0030 742.036; Eppendorf, Hamburg, Germany). We washed the cells twice with PBS, fixed them with 3.6% PFA in PBS for 20 min, permeabilized them with 0.2% Triton X-100 for 3 min, and blocked them with 1% BSA and 0.02% Triton X-100 in PBS for 1 h each at room temperature. Next, the cells were probed with the respective primary antibodies overnight at 4 °C and incubated with the respective secondary antibodies for 1 h at room temperature. Antibody information is available in Supplementary Table S1. The cells were washed thrice with PBS, and coverslips were mounted with SlowFade Gold antifade reagent (Invitrogen) or SlowFade Diamond antifade reagent (Invitrogen). For N-STORM analysis, we filled the cell chambers with the STORM imaging buffer with MEA prepared according to the manufacturer’s instructions. Next, we obtained images using an A1 + confocal laser microscope (Nikon), an N-STORM super-resolution microscope (Nikon), or an LSM 980 with Airyscan 2 (ZEISS, Oberkochen, Germany).

### Statistical analysis

The statistical significance of differences was analyzed using Dunnett’s test (Fig. 5b), Steel–Dwass test (Figs. 3b and 3c and Supplementary Figs. S8, S9b, and S15) as a multiple-comparison test, and data between the two groups were evaluated using Welch’s *t*-test (Figs. 2c and 4b and Supplementary Figs. S6 and S13). Cohen’s *d* was calculated as an effect size. To calculate Pearson’s coefficient for each image, we performed co-localization analysis using NIS-elements software (v4.2, Nikon) or Coloc2 (ImageJ Fiji software, https://imagej.net/Fiji.html#Downloads*)*. Values were expressed as the mean ± standard deviation (SD), and *P* < 0.05 was considered statistically significant. N.S., not significant.

## Supplementary Information

Supplementary Information

Supplementary Movie S1.

Supplementary Movie S2.
