# Supplementary material for: Applications of cell resealing to reconstitute microRNA loading to extracellular vesicles
Source: Sci Rep. 2021 Feb 3;11:2900. doi: 10.1038/s41598-021-82452-5 (PMC7859222; doi:10.1038/s41598-021-82452-5)
Supplement: Supplementary file 1 — Supplementary Information [file 41598_2021_82452_MOESM1_ESM.pdf]

Supplementary Information

Applications of cell resealing to reconstitute microRNA loading to extracellular vesicles

Yuki Sonoda[1], Fumi Kano[2], and Masayuki Murata[1, 2*]

[1] Department of Life Sciences, Graduate School of Arts and Sciences, The University of Tokyo, 3-8-1 Komaba, Meguro-ku, Tokyo, 153-8902, Japan

[2] Cell Biology Center, Institute of Innovative Research, Tokyo Institute of Technology, 4259 Nagatsuta, Midori-ku, Yokohama, Kanagawa, 226-8503, Japan

* Correspondence should be addressed to Masayuki Murata (email: mmurata@bio.c.u-tokyo.ac.jp)

INDEX

1 **Supplementary Figure legends**............2

2 **Supplementary Movies**............23

3 **Supplementary Tables**............23

4 **Supplementary Methods**............24

5 **Supplementary References**............26

Supplementary Figures

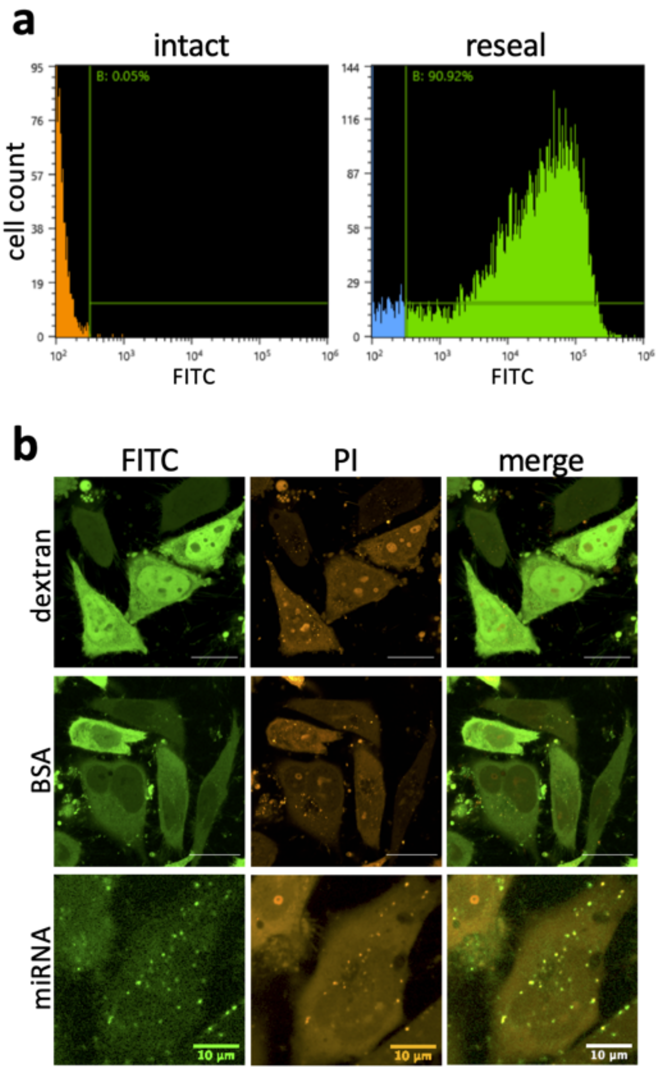

Supplementary Figure S1. Flow cytometric and morphometric analyses of resealed HeLa cells containing fluorescently labeled molecules.

(a) Flow cytometric analysis was performed for resealed HeLa cells. After introducing cytosol containing 100 μg/mL of 10 kDa fluorescein isothiocyanate-labeled dextran (FITC-dex), HeLa cells were incubated in a 5% CO_2 incubator for 120 min at 37°C and subjected to flow cytometry. The resealing efficiency was ~90%, indicating the cells succeeded in replacing cytosolic factors. (b) Fluorescence microscopy images of various fluorescently labeled molecules in resealed HeLa cells. During a pore-forming step induced by streptolysin O (SLO), the cells were stained with propidium iodide (PI).

After introducing cytosol containing 100 μg/mL of 10 kDa FITC-dex, 100 μg/mL of fluorescein isothiocyanate-labeled bovine serum albumin (FITC-BSA), or 1.0 μM fluorescein-labeled scrambled miRNA (SMC-4001), the resealed HeLa cells were incubated in a 5% CO_2 incubator for 120 min at 37°C. The culture medium (DMEM with phenol red) was changed with a fresh one (DMEM without phenol red), and the cells were observed at 37°C (live-cell imaging). Scale bar without text = 20 μm.

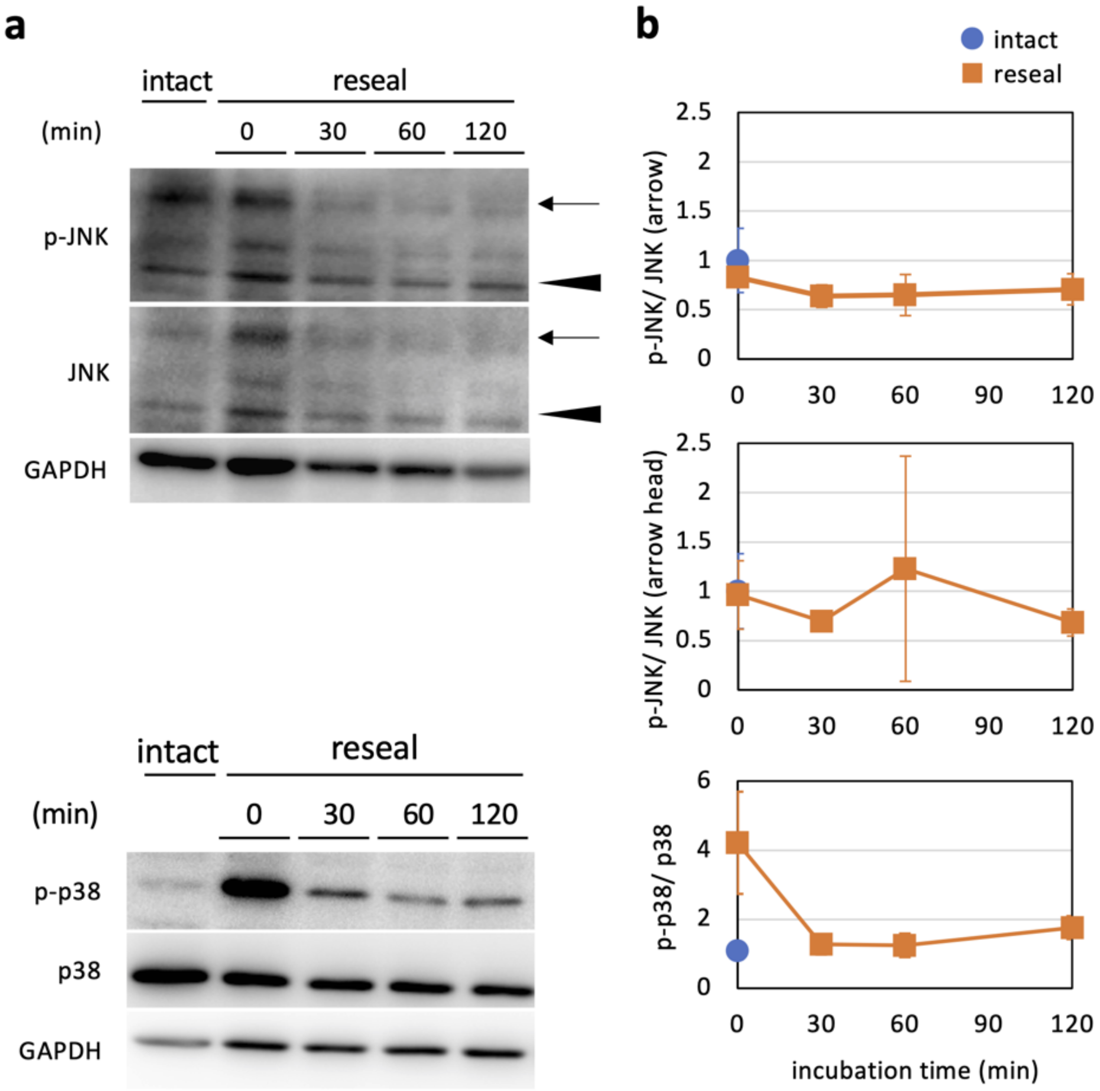

Supplementary Figure S2. Phosphorylation status of JNK and p38 MAPK in resealed HeLa cells.

(a) Resealed HeLa cells were incubated with DMEM (+fetal calf serum, FCS) in a 5% CO_2 incubator for 0, 30, 60, and 120 min at 37°C. Intact or resealed HeLa cells were lysed and subjected to western blotting (WB) analysis using antibodies against c-Jun N-terminal kinase (JNK), phosphorylated JNK (p-JNK), p38 mitogen-activated protein kinase (MAPK), and phosphorylated p38 MAPK (p-p38). Arrows indicate estimated JNK bands at ~54 kDa. Arrowheads indicate estimated JNK bands at ~46 kDa. (b) Bands for p-JNK, JNK, p-p38, p-38, and glyceraldehyde 3-phosphate dehydrogenase

(GAPDH) in intact (●) and resealed (■) HeLa cells were quantified, and p-JNK–JNK and p-p38–p38 mean ratios were calculated, normalized by GAPDH. Data represent results from three independent experiments ($n = 3$), expressed as the mean ± standard deviation (SD).

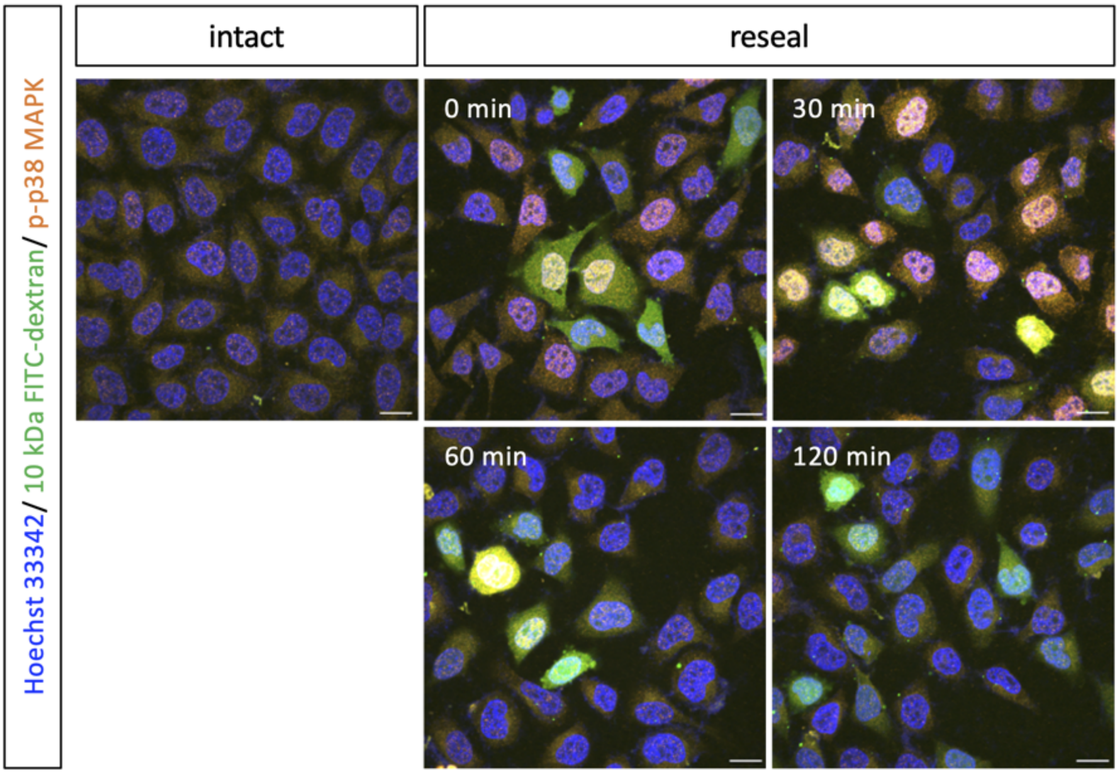

Supplementary Figure S3. Time-dependent subcellular localization changes of phosphorylated p38 MAPK in resealed HeLa cells.

After introducing cytosol containing 100 µg/mL of 10 kDa FITC-dex, resealed HeLa cells were incubated with DMEM (+FCS) in a 5% CO_2 incubator for 0, 30, 60, and 120 min at 37°C. Intact or resealed HeLa cells were fixed and subjected to immunofluorescence (IF) analysis using anti-phosphorylated p38 MAPK antibody. At 60 min after resealing, the fluorescence signal of p-p38 MAPK in the nucleus decreased, indicating that a diminished cellular stress response occurred within ~60 min. Scale bar = 20 µm.

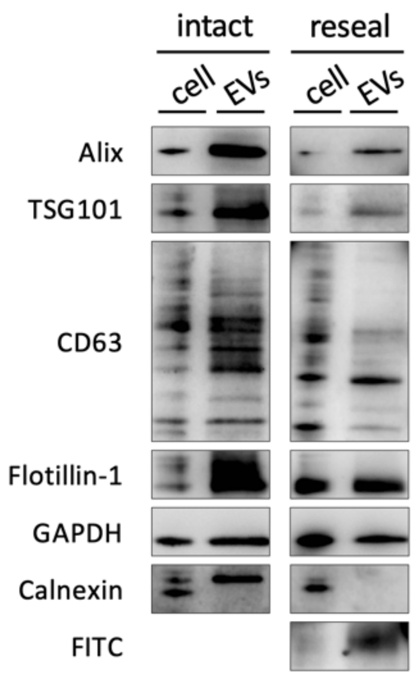

Supplementary Figure S4. Biochemical characterization of EVs derived from resealed H4IIEC3 cells.

WB analysis was performed for whole cells and extracellular vesicles (EVs) derived from resealed H4IIEC3 cells. We obtained whole-cell lysates and EVs by ultracentrifugation method (UC EVs), as described in Fig. 1d. Estimated bands of exogenously added FITC-BSA were clearly observed in EVs but slightly in cells relative to those in Fig. 1d, indicating that BSA cellular metabolism and exocytosis in hepatocytes might be more rapid compared to epithelial-like HeLa cells.

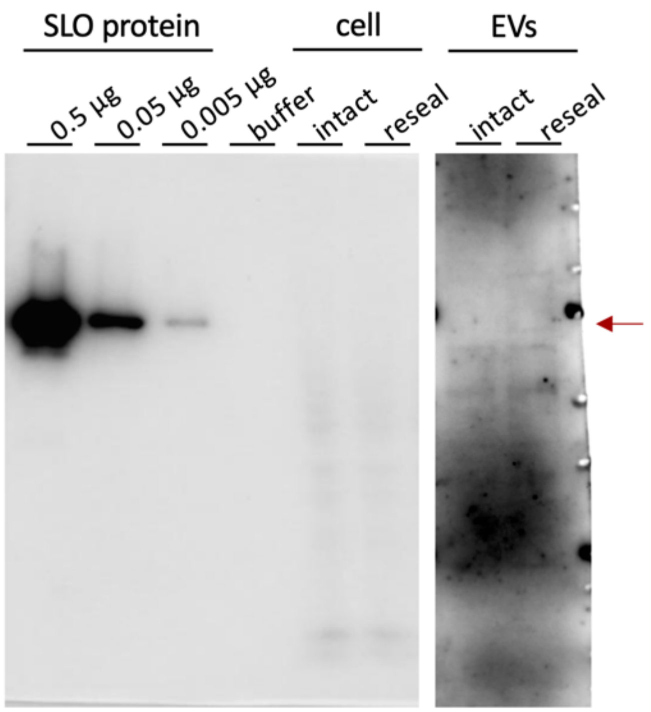

Supplementary Figure S5. Non-existence of SLO-induced bleb or microvesicles in EVs derived from resealed HeLa cells.

WB analysis using anti-SLO antibody was performed for whole cells and UC EVs, as described in Fig. 1d. Respective amounts of recombinant proteins (SLO) were loaded to lanes and subjected to WB analysis using anti-SLO antibody. The buffer indicates control for SLO lanes. The arrow indicates the estimated SLO band position.

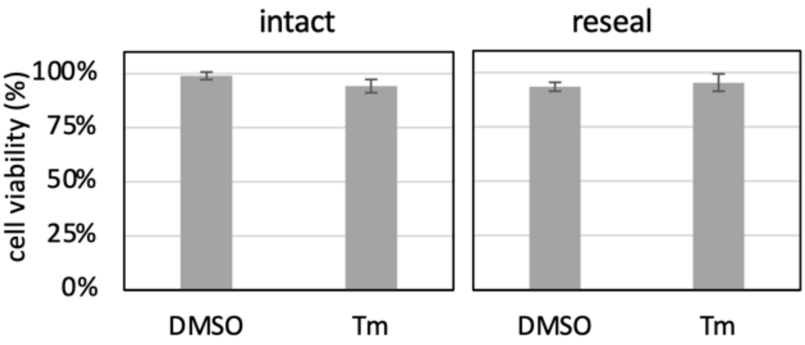

Supplementary Figure S6. Cell viability assay of resealed HeLa cells related to Fig. 1e.

Quantification of cell viability in the presence or absence of the endoplasmic reticulum stressor tunicamycin (Tm), as described in Fig. 1e. Resealed HeLa cells were trypsinized, collected in DMEM (+FCS), and examined by the trypan blue dye exclusion test. Data represent results from three independent experiments (*n* = 3), expressed as the mean ± SD. There was no significant difference between dimethyl sulfoxide (DMSO) and Tm treatment on Welch’s *t*-test.

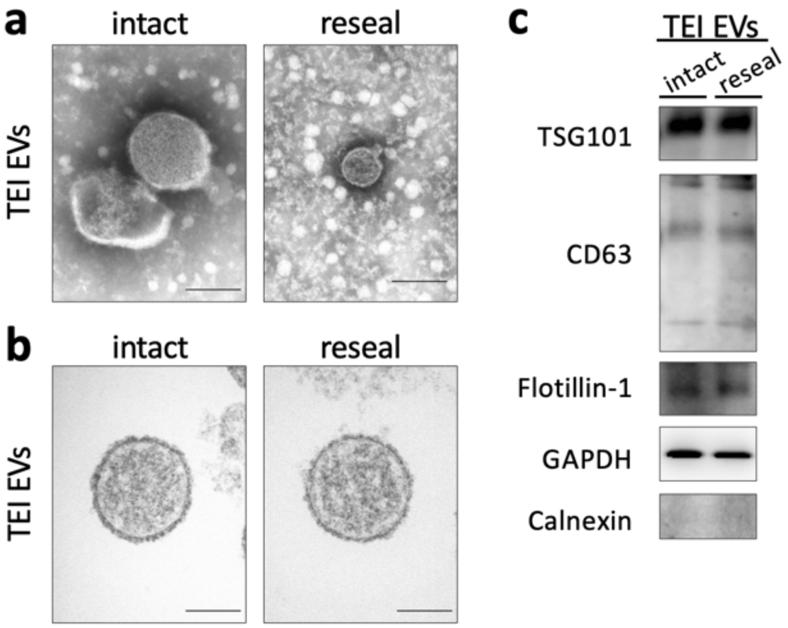

Supplementary Figure S7. Characterization of HeLa cell-derived EVs obtained using Total Exosomes Isolation reagent.

(a) Resealed HeLa cells in which L5178Y cytosol was introduced were incubated in a 5% CO_2 incubator for 120 min at 37°C, and the medium was replaced with fresh medium. The cells were further incubated in a 5% CO_2 incubator for 48 h at 37°C, and EVs were obtained using Total Exosomes Isolation reagent (TEI EVs) and subjected to negative-stained electron microscopy (EM). Scale bar = 100 nm. (b) TEI EVs were prepared, as described in (a), and subjected to EM using a chemical fixation method. Scale bar = 100 nm. (c) WB analysis was performed for whole cells and TEI EVs obtained as described in (a).

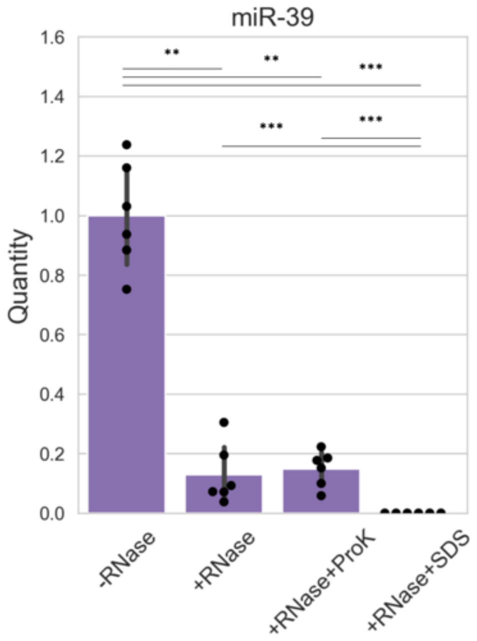

Supplementary Figure S8. Quantification of exogenously added miRNA encapsulated in EVs derived from resealed HeLa cells.

Quantification of the exogenously added miRNA in EVs derived from resealed HeLa cells under various conditions. Resealed HeLa cells in which L5178Y cytosol containing a 1.0 nM synthesized cel-miR-39 was introduced were incubated in a 5% CO_2 incubator for 120 min at 37°C (post-incubation), and the medium was replaced with fresh medium. The cells were further incubated in a 5% CO_2 incubator for 48 h at 37°C, and TEI EVs were obtained and subjected to the RNase protection assay. Total EV-RNA was extracted, and miR-39-3p levels were measured by real-time PCR using the TaqMan assay. Quantification was performed using the standard curve method. Values were normalized by the mean values of "−RNase" control. Substantial extracellular miRNA levels remained even in the presence of RNase. These RNase-resistant miRNAs were poorly degraded by proteinase K (ProK) treatment in the presence of RNase, and completely degraded only when the membrane components were disrupted by sodium dodecyl sulfate (SDS) treatment in the presence of RNase. These results suggested that RNase-resistant miRNAs were encapsulated in TEI EVs.

Data represent results from six independent experiments ($n = 6$), expressed as the mean ± SD. $^{**}P < 0.01$; $^{***}P < 0.001$.

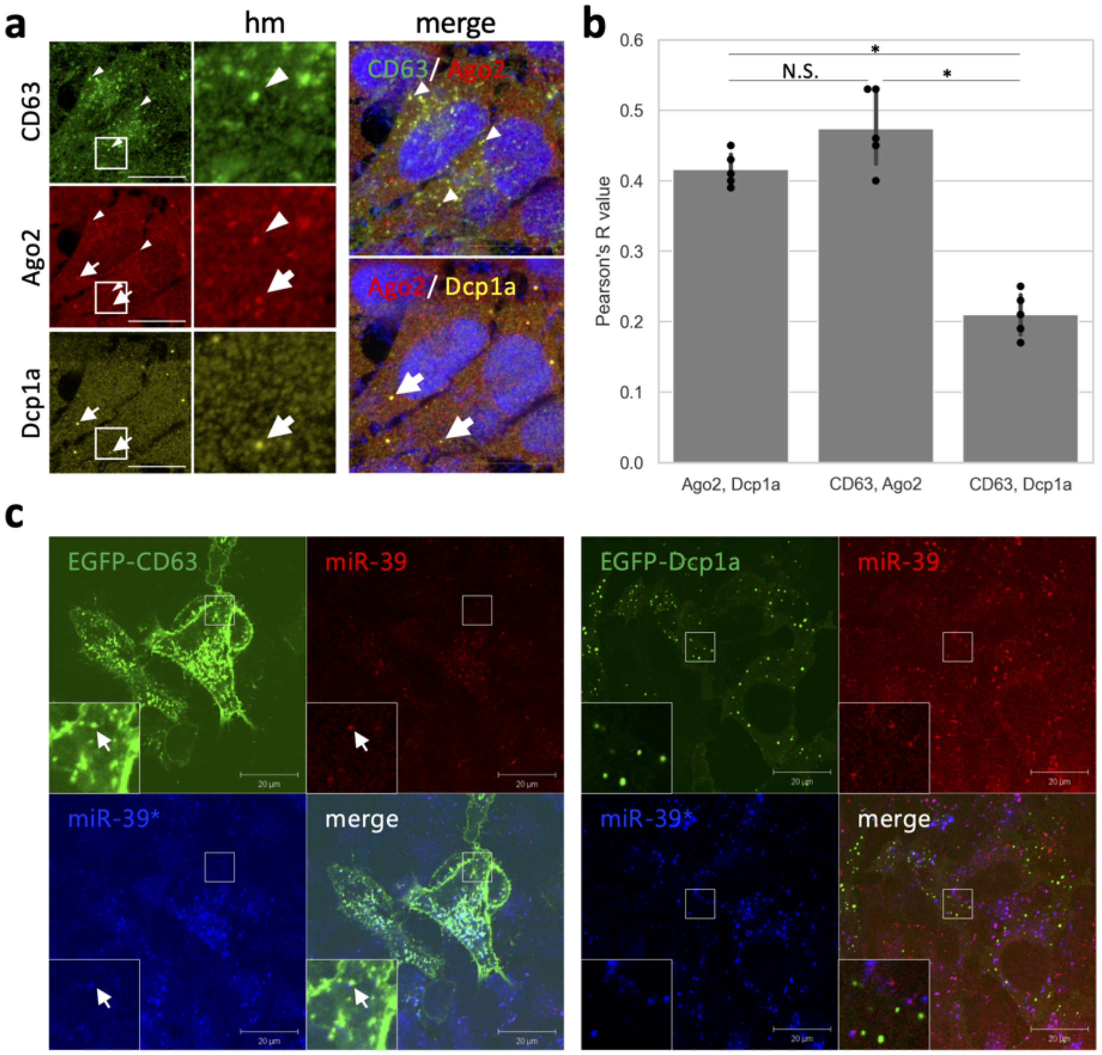

Supplementary Figure S9. Localization of Ago2 and miRNA to CD63-positive MVEs or Dcp1a-positive P-bodies in HeLa cells.

(a) Fluorescence microscopy images of Argonaute 2 (Ago2) to CD63 and Dcp1a in intact HeLa cells. HeLa cells were subjected to IF analysis using anti-CD63, anti-Ago2, and anti-Dcp1a antibodies and then observed using confocal microscopy. Ago2 was co-localized with both CD63-positive multivesicular endosomes (MVEs) and Dcp1a-positive P-bodies. CD63-positive MVEs and Dcp1a-positive P-bodies were not co-localized. Arrows indicate Ago2-positive spots co-localized with Dcp1a. Arrowheads indicate Ago2-positive spots co-localized with CD63. Scale bar = 20 µm. (b) Bar plots show Pearson’s correlations on the basis of the triple-stained image. Data represent

results from five frames ($n = 5$), expressed as the mean ± SD. *$P < 0.05$. (c) Fluorescence microscopy images of transiently expressed CD63-EGFP or Dcp1a-EGFP in resealed HeLa cells. HeLa cells were transfected with the EGFP-CD63 or the EGFP-Dcp1a vector by lipofection and further incubated in a 5% CO_2 incubator for 2 days at 37°C. After introducing cytosol containing a 1.5 μM fluorescently labeled double-stranded miRNA mimic (Cy3 for cel-mir-39, guide strand; Cy5 for cel-mir-39*, passenger strand), the cells were post-incubated for 120 min, fixed, and observed using confocal microscopy. miRNA was co-localized only with CD63-positive MVEs but not Dcp1a-positive P-bodies, indicating accumulation of cytosolic miRNAs to MVEs but not to P-bodies. Arrows indicate miRNAs co-localized with CD63-positive MVEs. Scale bar = 20 μm.

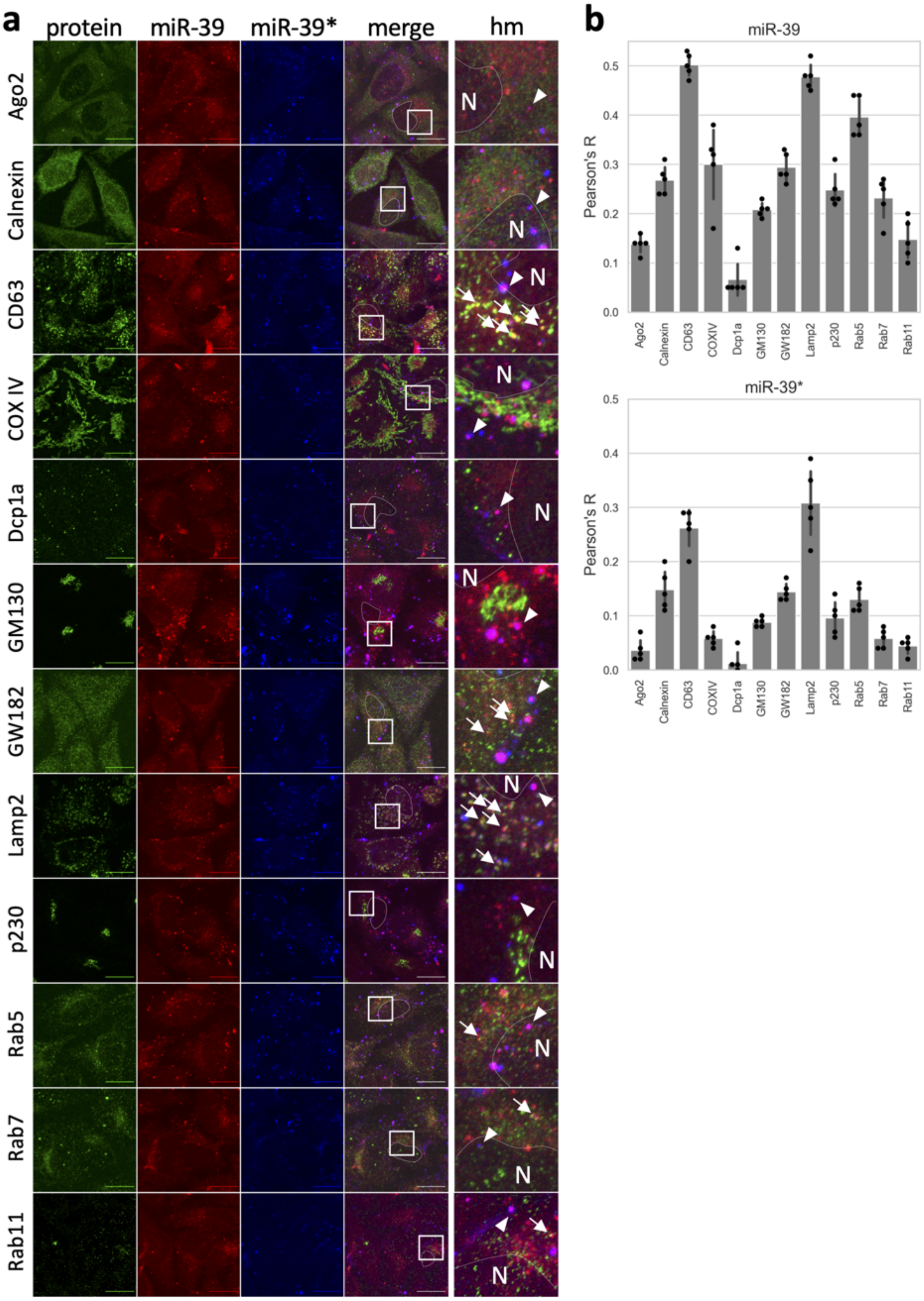

Supplementary Figure S10. Subcellular localization of fluorescently labeled miRNA mimic introduced into resealed HeLa cells.

(a) Fluorescence microscopy images of miRNA introduced into resealed HeLa cells.

After introducing cytosol containing 1.5 μM fluorescently labeled miRNA, resealed HeLa cells were post-incubated for 120 min and subjected to IF analysis using respective antibodies. Guide strands substantially localized to CD63 (MVEs) and Lamp2 (lysosomes). They weakly localized to endosome-related proteins Rab7 (late endosomes) and Rab11 (recycling endosomes). The observed guide and passenger strand double-positive spots might indicate double-stranded miRNA. Right panels are a magnified image of the white boxes in left panels. Nuclear regions are indicated as "N" inside the dotted line. Arrows indicate the target protein co-localized with the guide strand. Arrowheads indicate guide and passenger strand double-positive spots. Scale bar = 20 μm. hm: high magnification. (b) Bar plots show Pearson's correlations. Data represent results from five frames (n = 5), expressed as the mean ± SD.

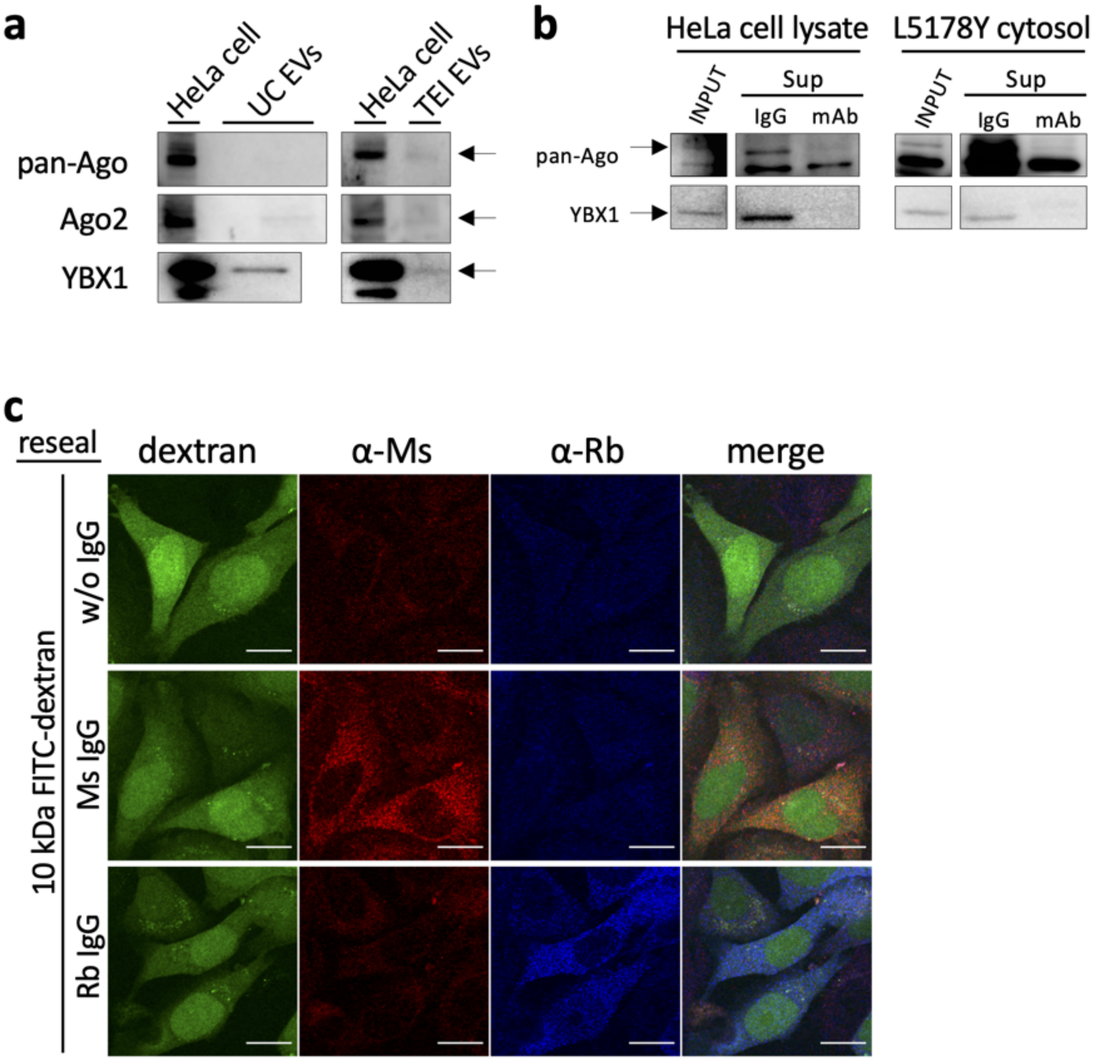

Supplementary Figure S11. Biochemical and morphological analyses of antibody capacity for recognition, interaction, and resealing in HeLa cells and the L5178Y cytosol.

(a) WB analysis using anti-pan- Ago, anti-Ago2, and anti-Y-box binding protein 1 (YBX1) antibodies was performed for intact HeLa cells and EVs derived from intact HeLa cells. Both proteins were detected in HeLa cell lysate as bands with the estimated molecular weight. Only YBX1 protein was detected in EVs from HeLa cells. Arrows indicate the estimated position of target bands. UC EVs: EVs obtained via differential ultracentrifugation. TEI EVs: EVs obtained using Total Exosome Isolation reagent. (b) Confirmation of epitope-recognition and interaction capability of antibodies. To analyze

the epitope-recognition and interaction capability of antibodies, immunoprecipitation assay was performed using anti-Ago and anti-YBX1 antibodies. Immunodepletion of pan-Ago or YBX1 protein in HeLa cell lysate or L5178Y cytosol was successfully accomplished by using the corresponding antibodies. Ten percent of the input was also loaded. Arrows indicate the estimated position of target bands. mAb, monoclonal antibody; IgG, immunoglobulin G. (c) Fluorescence microscopy images of introduced antibodies in resealed HeLa cells. After introducing cytosol containing 10 μg/mL of normal IgG and 100 μg/mL of 10 kDa FITC-dex, the resealed HeLa cells were incubated in a 5% CO_2 incubator for 12 h at 37°C and subjected to IF analysis using Alexa Fluor 546-conjugated mouse antibody (α-Ms) and Alexa Fluor 647-conjugated anti-rabbit antibody rabbit (α-Rb). Both normal IgG of mouse (Ms IgG) and rabbit (Rb IgG) were detected. Scale bar = 20 μm.

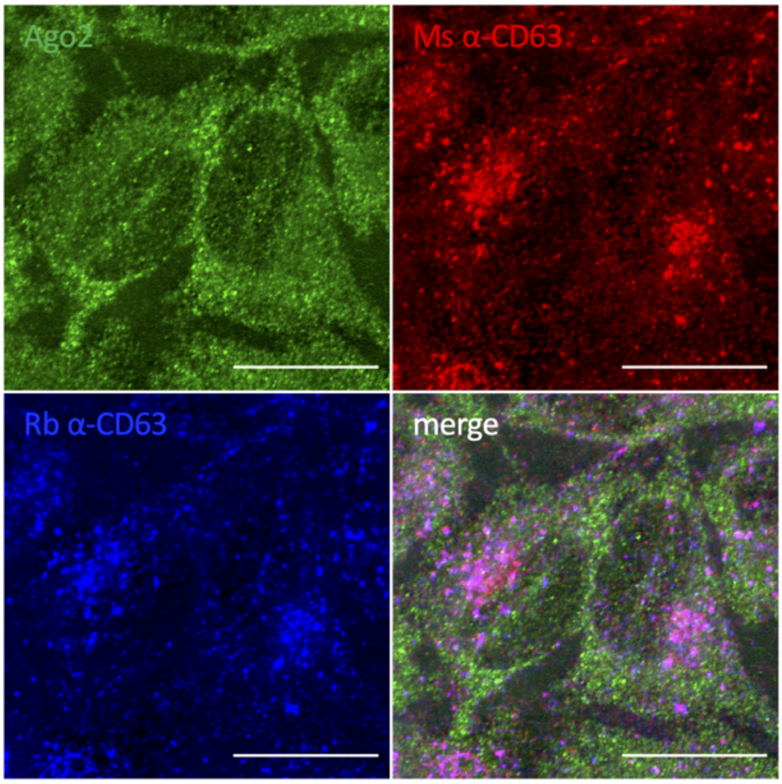

Supplementary Figure S12. Observation of CD63-positive MVEs using two different antibodies against CD63 in HeLa cells.

Fluorescent microscopy images of CD63-positive MVEs in intact HeLa cells. HeLa cells were subjected to IF analysis using mouse anti-CD63 antibody (Ms α-CD63), rabbit anti-CD63 antibody (Rb α-CD63), and anti-Ago2 antibody, and were observed using confocal microscopy. Mouse and rabbit anti-CD63 antibodies showed the overlapped staining in HeLa cells. Scale bar = 20 μm.

a

No.	antibody resealed	miR-122 cell count	miR-122 *p*-value	miR-122 Cohen's *d*	miR-34a cell count	miR-34a *p*-value	miR-34a Cohen's *d*	miR-423-3p cell count	miR-423-3p *p*-value	miR-423-3p Cohen's *d*
1	normal IgG	147	6.58E-19	1.045	147	1.14E-08	0.658	161	0.0155	0.264
2	anti-pan-Ago mAb	184			172			179		
3	normal IgG	176	9.80E-16	1.001	161	6.03E-06	0.500	209	0.822	0.0249
4	anti-YBX1 mAb	122			178			134		

b

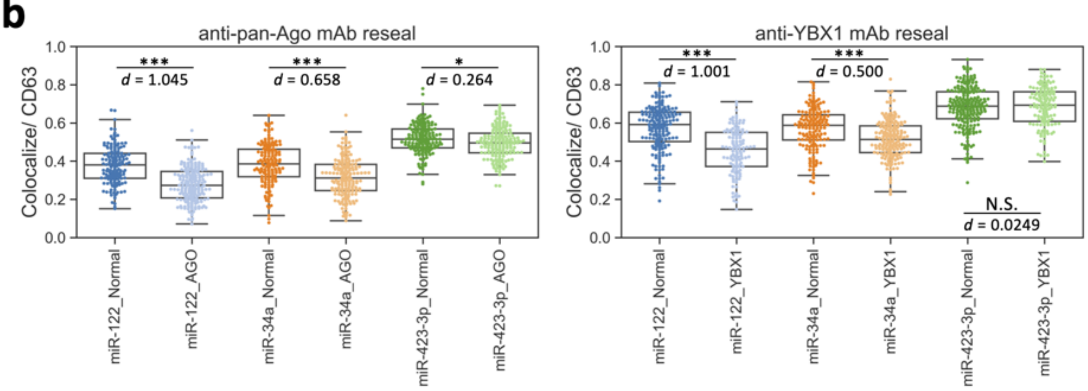

Supplementary Figure S13. Quantification of the co-localization ratio of miRNA and CD63-positive MVEs in resealed HeLa cells containing function-blocking antibodies.

(a) The table shows the number of cells determined using image-based quantification (Fig. 4e). As described in Fig. 4, we detected the cell areas and counted the number of cells automatically. (b) Box plots show the quantification results of the co-localization ratio of miRNA and CD63-positive MVEs. To calculate the co-localization ratio in each cell, we divided the number of co-localized dots (Fig. 4e, y-axis) by the number of CD63 dots (Fig. 4e, x-axis). *$P < 0.05$; ***$P < 0.001$. We also calculated and indicated the values of Cohen’s *d* as an effect size.

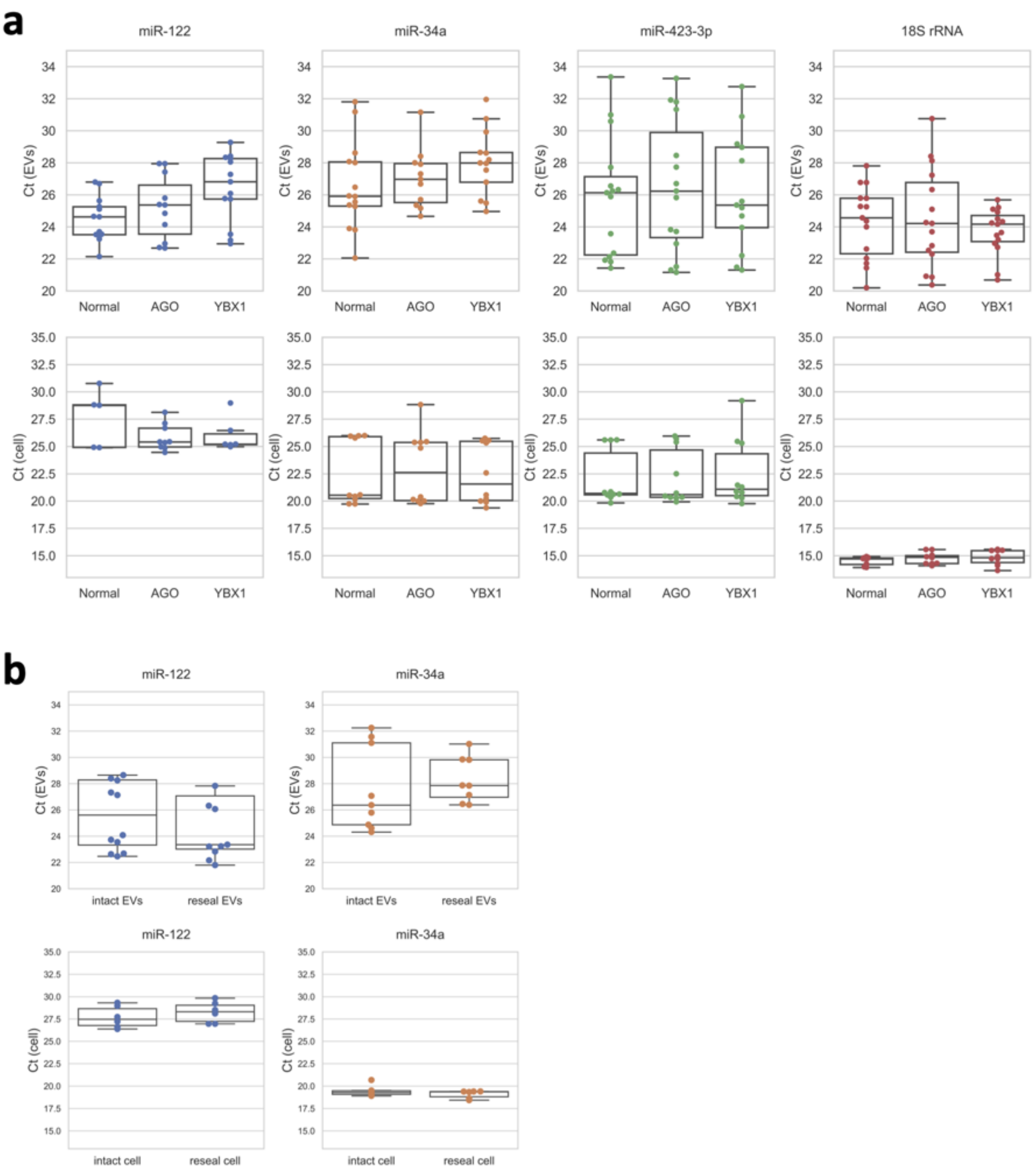

Supplementary Figure S14. Quantitative analysis of miRNAs in isolated EVs derived from resealed HeLa cells related to Fig. 5.

(a) Raw Ct values from Fig. 5a. (b) Quantification of the endogenously expressed RNA amount in whole cells and EVs obtained 120 min after resealing (post-incubation). Intact and resealed HeLa cells were post-incubated and miR-122 and miR-34a amounts were measured by real-time PCR. Box plots show raw Ct values of cellular- and EV-miRNA. intact EVs, n = 12; reseal EVs, n = 11; intact cell, n = 6; reseal cell, n = 6.

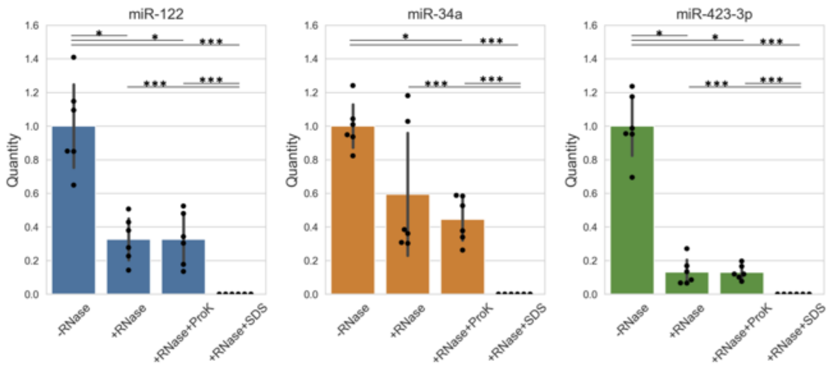

Supplementary Figure S15. Quantification of endogenously expressed miRNA encapsulated in EVs derived from resealed HeLa cells.

Quantification of the endogenously expressed miRNA in EVs derived from resealed HeLa cells under various conditions. TEI EVs were collected from resealed HeLa cells, as described in Supplementary Fig. S8, and subjected to the RNase protection assay. Total EV-RNA was extracted, and miR-122, miR-34a, and miR-423-3p levels were measured by real-time PCR using the TaqMan assay. Quantification was performed using the standard curve method. Values were normalized by the mean values of the "−RNase" control. As described in Supplementary Fig. S8, we detected RNase-resistant miRNAs, which were degraded completely by SDS treatment in the presence of RNase. These results suggested that endogenously expressed miRNAs were encapsulated in TEI EVs. Data represent results from six independent experiments (n = 6), expressed as the mean ± SD. *$P < 0.05$; ***$P < 0.001$.

Supplementary Movies

Supplementary Movie S1. Live-cell imaging of EGFP-CD63-stably expressing HeLa cells.

Intact and resealed HeLa cells were incubated in a 5% CO_2 incubator for 120 min at 37°C and then observed using LSM980 with Airyscan2. Scale bar = 20 μm. The playback speed was set to 20×.

Supplementary Movie S2. High-magnification videos from Supplementary Movie S1.

Supplementary Movie S1 was magnified. The 98th image of intact HeLa cells and the 405th image of resealed HeLa cells are indicated in Fig. 2b. Scale bar = 500 nm.

Supplementary Tables

Supplementary Table S1. Antibodies used for immunofluorescence or immunoblotting.

Supplementary Table S2. Primers used for real-time PCR.

Supplementary Methods

Plasmid construction and transfection

To synthesize the EGFP-CD63 expression construct, we amplified a cDNA fragment for CD63 from HeLa cell lysates by PCR with the following oligonucleotide pairs (5'-ATGGCGGTGGAAGGAGGAATGA-3', 5'-CTACATCACCTCGTAGCCACTTCTGAT -3'). Next, we inserted the fragment into the SmaI site in the pEGFP-C2 vector (Clontech Laboratories, Mountain View, CA, USA). The EGFP-Dcp1a construction was as described previously[1].

Transient plasmid transfections were performed using the FuGENE HD Transfection Reagent (Promega), and the transfectants were cultured for 2 days. To obtain a stable cell line, we cultured the cells for at least 2 weeks at a concentration of 800 ng/mL G418. Next, we selected single colonized cells. siRNA transfection was performed using RNAiMAX (Invitrogen), and the transfectants were cultured for 18 h. All experiments were performed according to the manufacturer's instructions.

Flow cytometry

HeLa cells were washed twice with PBS, trypsinized, and collected in DMEM (+FCS). The cells were then subjected to flow cytometry using an SH800 cell sorter (Sony Corporation, Tokyo, Japan).

Chemical fixation for electron microscopy.

According to the previously study[2], the samples were fixed with 2% glutaraldehyde in 0.1 M phosphate buffer (PB) pH 7.4 for 1 h at 4°C. After fixation, the samples were washed thrice with 0.1 M PB for 10 min each and post-fixed with 2% osmium tetroxide (OsO_4) in 0.1 M PB for 1 h at 4°C. Next, the samples were dehydrated in a graded

ethanol series (50%, 70%, 90%, and anhydrous). The schedule was as follows: 50% and 70% for 10 min each at 4°C, 90% at room temperature for 10 min, and anhydrous ethanol four times at room temperature for 10 min each. Next, the samples were infiltrated with propylene oxide (PO) twice for 10 min each and placed in a 7:3 mixture of PO and resin (Quetol-812; Nisshin EM, Tokyo, Japan) for 1 h. The tubes were left open, and PO was volatilized overnight. Next, the samples were transferred to fresh 100% resin and polymerized at 60°C for 48 h. After embedding, the polymerized resins were sectioned at 70 nm with a diamond knife using an ultramicrotome (Ultracut UCT; Leica, Vienna, Austria), and the sections were mounted on copper grids. They were stained with 2% uranyl acetate at room temperature for 15 min, and then washed with distilled water followed by secondary staining with Lead stain solution (Sigma-Aldrich, St. Louis, MO, USA) at room temperature for 3 min. Finally, the grids were observed under a JEM-1400 Plus transmission electron microscope (JEOL, Tokyo, Japan) at an acceleration voltage of 100 kV. Digital images (3296 × 2472 pixels) were captured with an EM-14830RUBY2 charged-coupled device camera (JEOL).

RNase protection assay

Extracellular vesicles obtained using Total Exosomes Isolation reagent in PBS were incubated with 5 μg/mL proteinase K (Takara Bio, Shiga, Japan) or PBS for 5 min at 37°C, followed by incubation with cOmplete EDTA-free protease inhibitor cocktail (Roche, five times concentrated) for 10 min on ice to inhibit proteinase K activity. Next, 10% SDS or PBS was added at a final concentration of 1% to each sample, and samples were incubated with 0.1 mg/mL RNase A (Millipore) or PBS for 15 min at 37°C. Finally, we extracted total RNA using the miRNeasy Mini Kit (QIAGEN) and stored it at −80°C.

Immunoprecipitation

HeLa cells were lysed with ice-cold radioimmunoprecipitation assay buffer containing cOmplete EDTA-free protease inhibitor cocktail and PhosSTOP (Roche) at the recommended concentration and then passed 15 times through a 27-gauge needle. Next, the samples were incubated with Protein G Sepharose 4 Fast Flow affinity resin (GE Healthcare) for 1 h at 4°C. After centrifugation at 12,000 ×*g* for 10 min, the supernatants were incubated overnight at 4°C with 50 µg/mL of the respective antibodies and then were incubated with affinity resin for 3 h at 4°C. After another centrifugation at 12,000 ×*g* for 1 min, we collected the immunoprecipitates, separated them by SDS-polyacrylamide gel electrophoresis, and then immunoblotted them with the respective antibodies.

Supplementary References

1. Fujimura, K., Kano, F. & Murata, M. Dual localization of the RNA binding protein CUGBP-1 to stress granule and perinucleolar compartment. *Exp. Cell Res.* **314**, 543–553 (2008).
2. Kuchitsu, Y., Homma, Y., Fujita, N. & Fukuda, M. Rab7 knockout unveils regulated autolysosome maturation induced by glutamine starvation. *J. Cell Sci.* **131**, jcs215442 (2018).

full-length-0001.tif

Figure. 1d and Figure. S7b

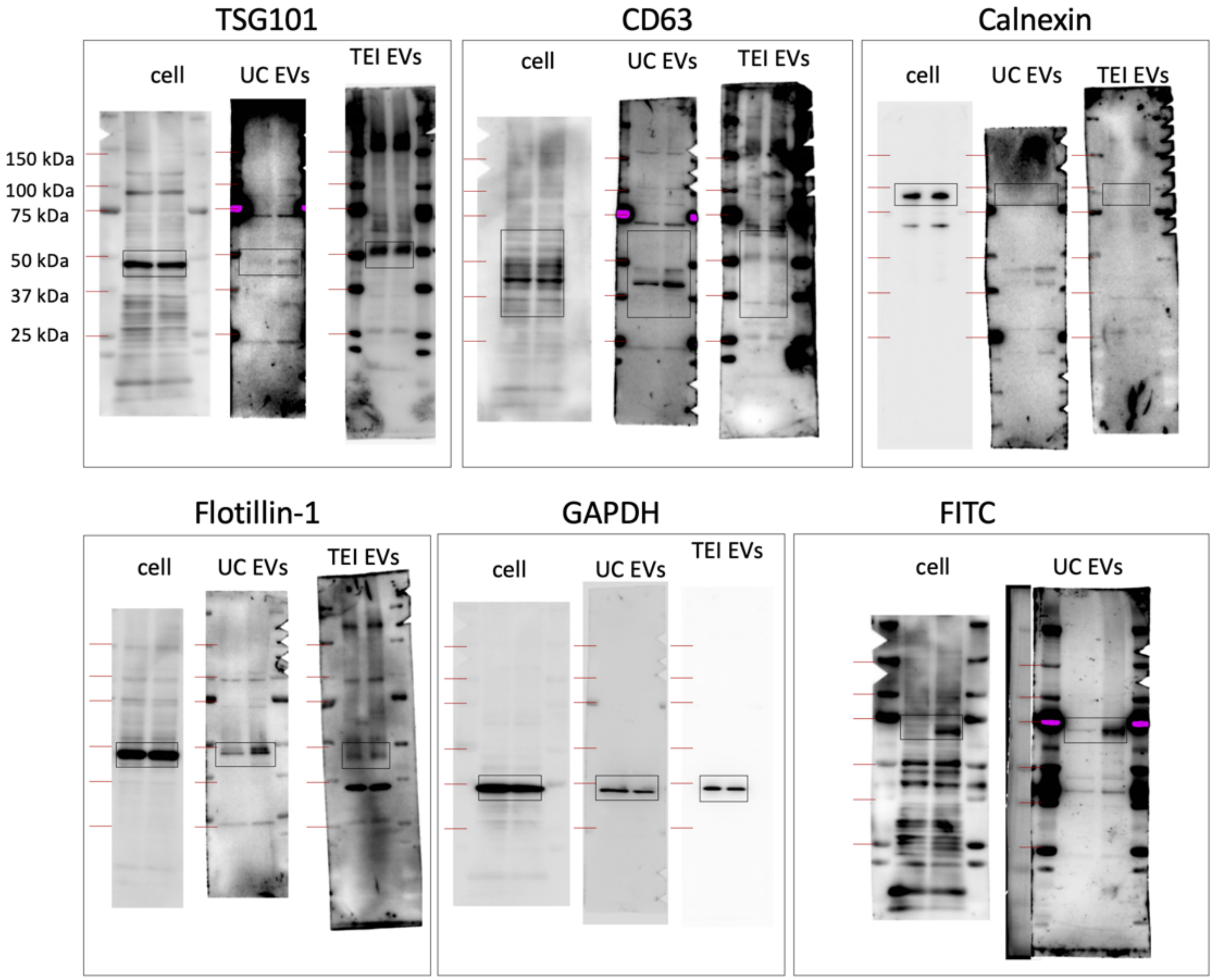

Supplementary Information

full-length-0002.tif

Figure. 4a

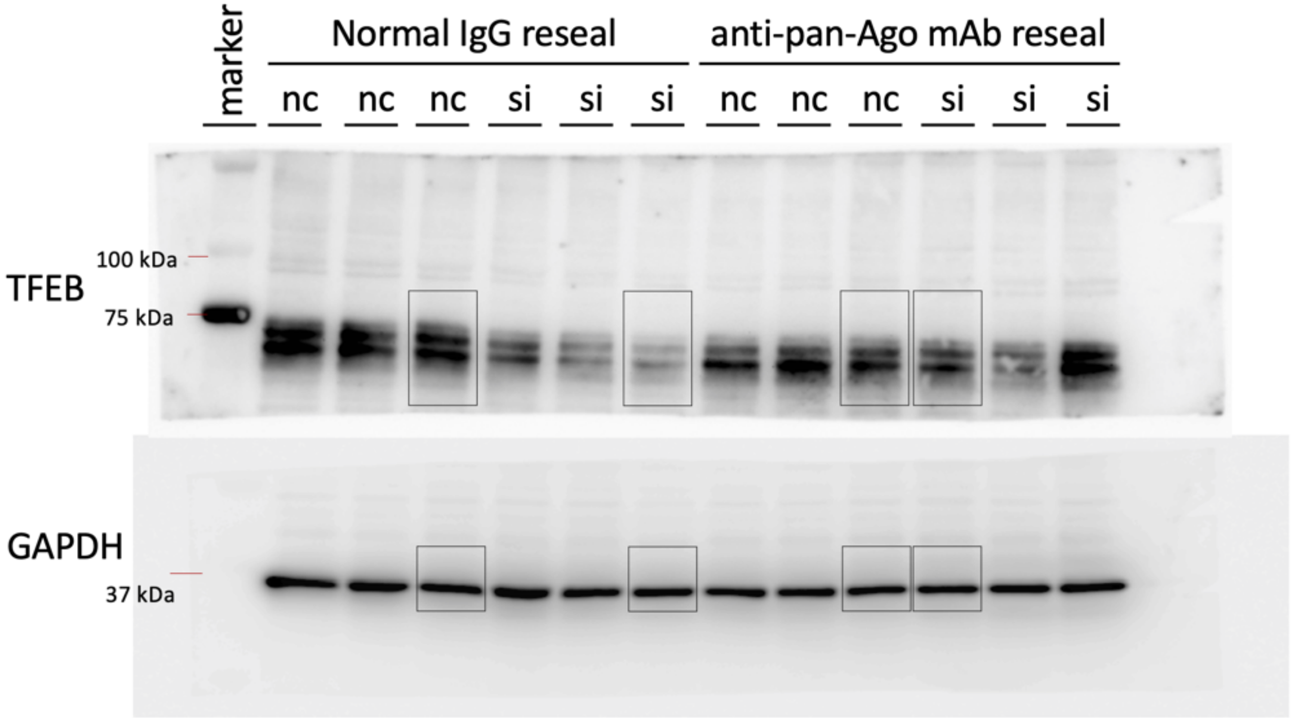

Supplementary Information

full-length-0003.tif

Supplementary Figure. S2a

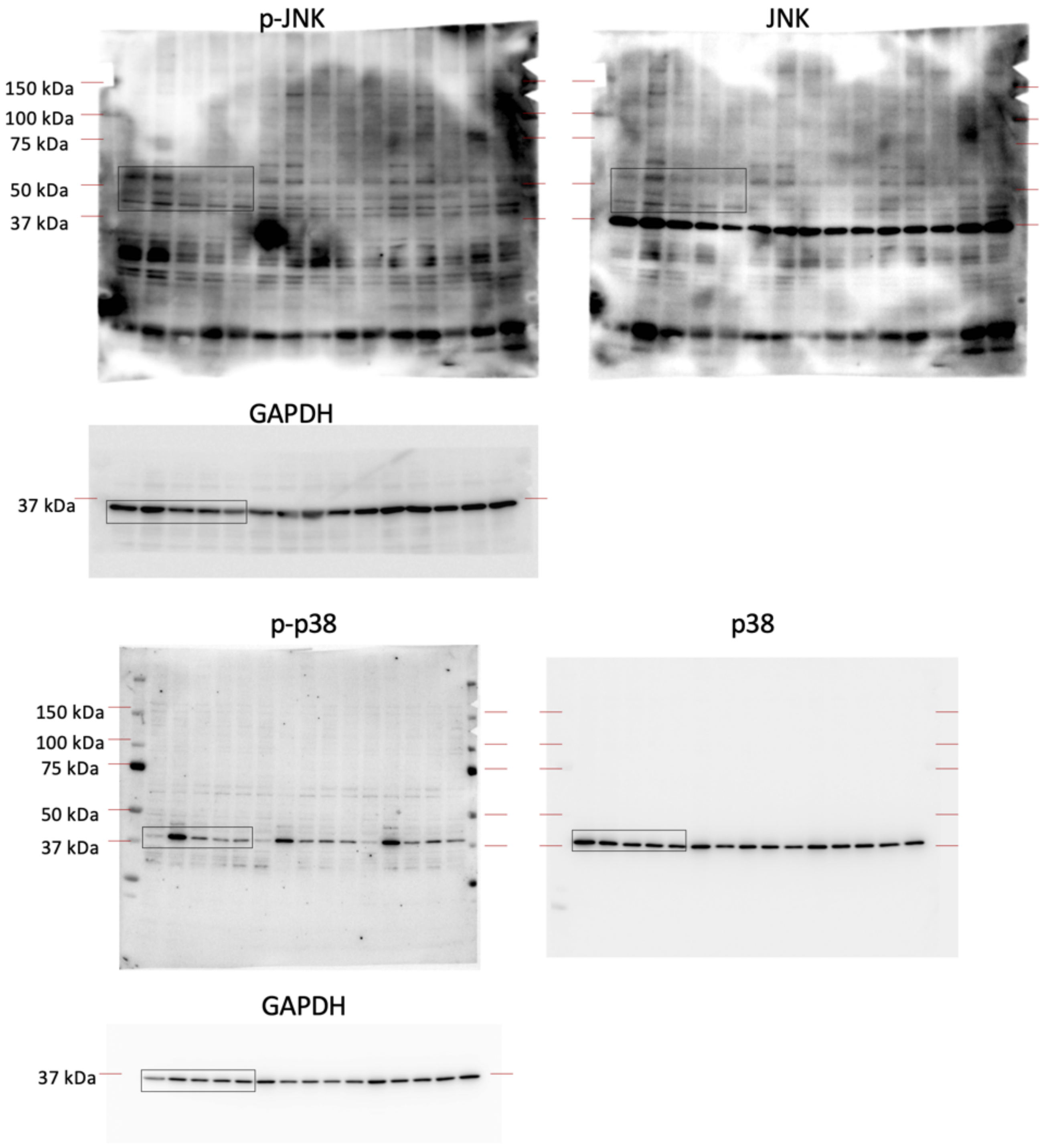

Supplementary Figure. S4

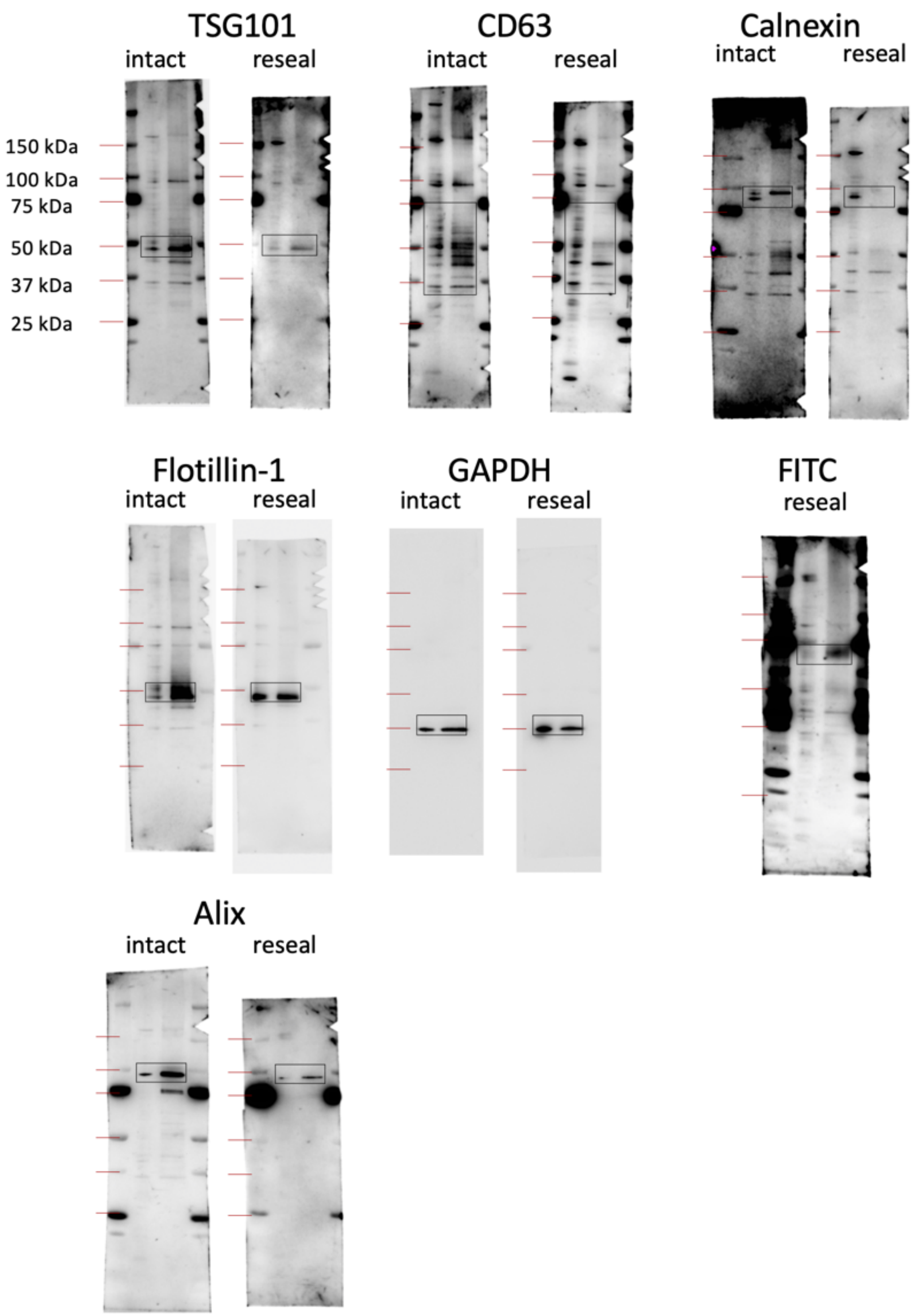

full-length-0005.tif

Supplementary Figure. S11a

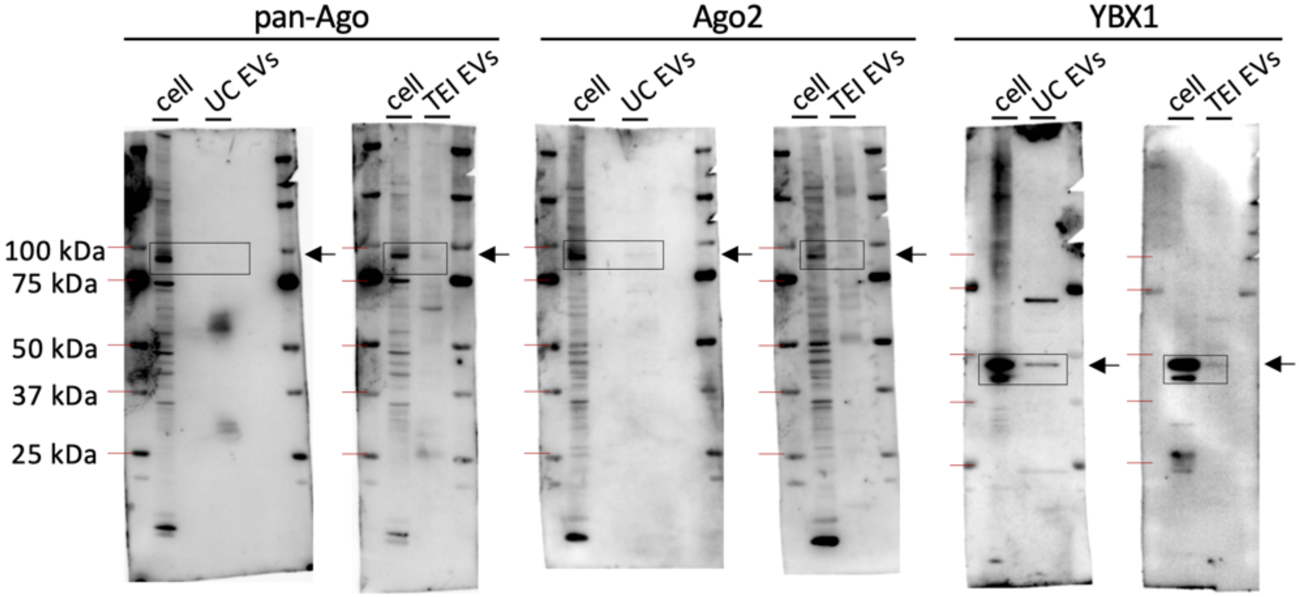

Supplementary Information

full-length-0006.tif

Supplementary Figure. S11b

Different exposures of the same membrane are grouped by black lines.
IP resultants and supernatants from lysates are shown as *"1st."*
IP resultants and supernatants from 1st supernatants are shown as *"2nd."*

HeLa Cell Lysate, anti-pan-Ago antibody

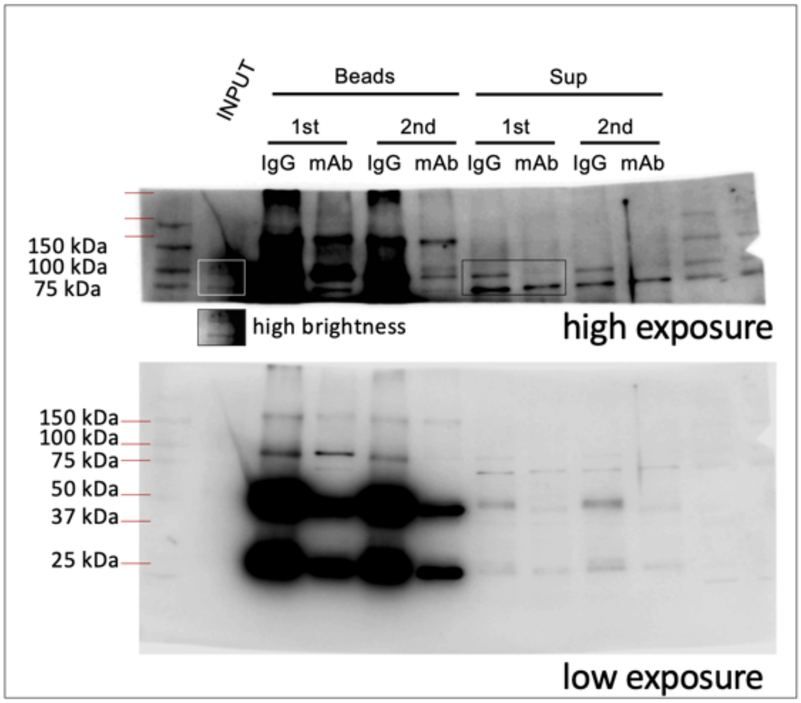

Western blotting

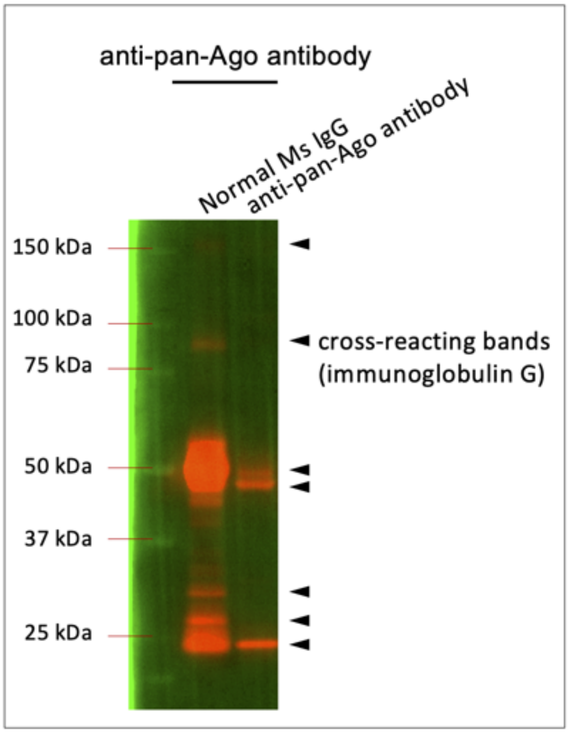

L5167Y Cytosol, anti-pan-Ago antibody

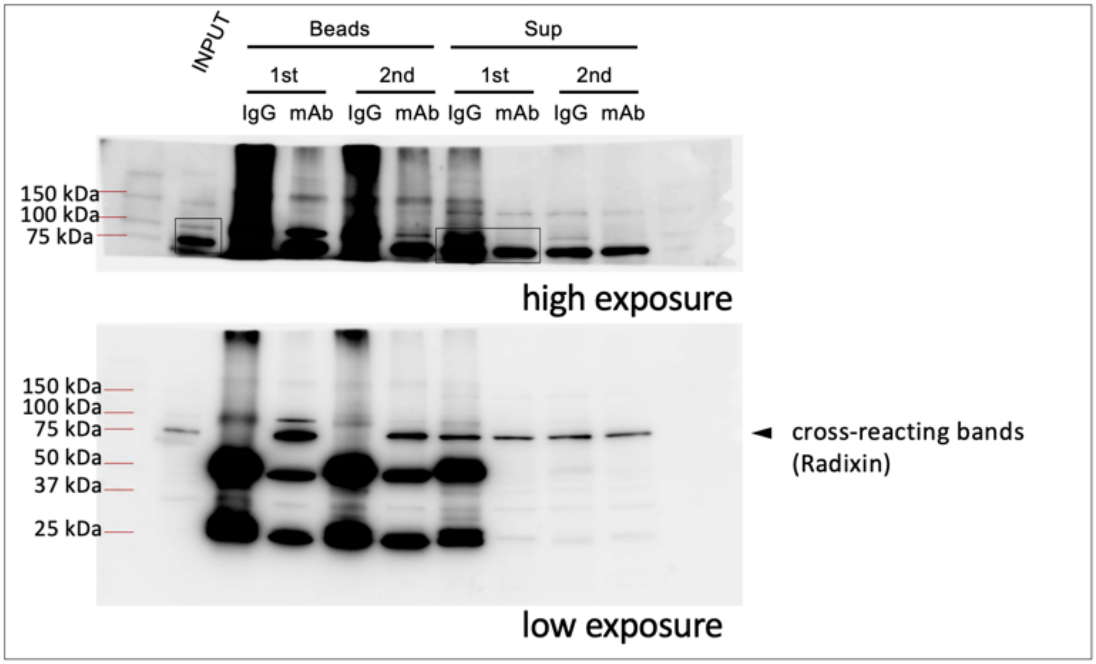

full-length-0007.tif

Supplementary Figure. S11b

IP resultants and supernatants from lysates are shown as *"1st."*
IP resultants and supernatants from 1st supernatants are shown as *"2nd."*

HeLa Cell Lysate, anti-YBX1 antibody

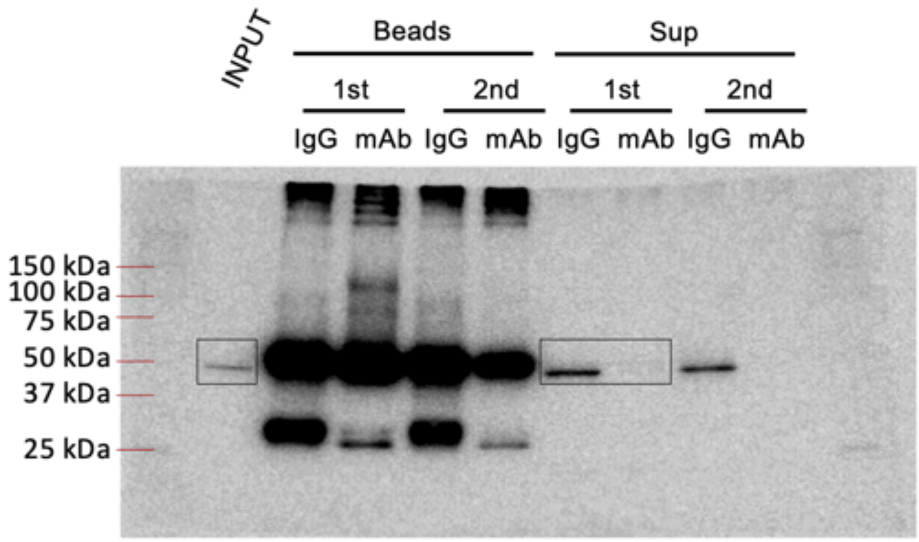

L5178Y Cytosol, anti-YBX1 antibody

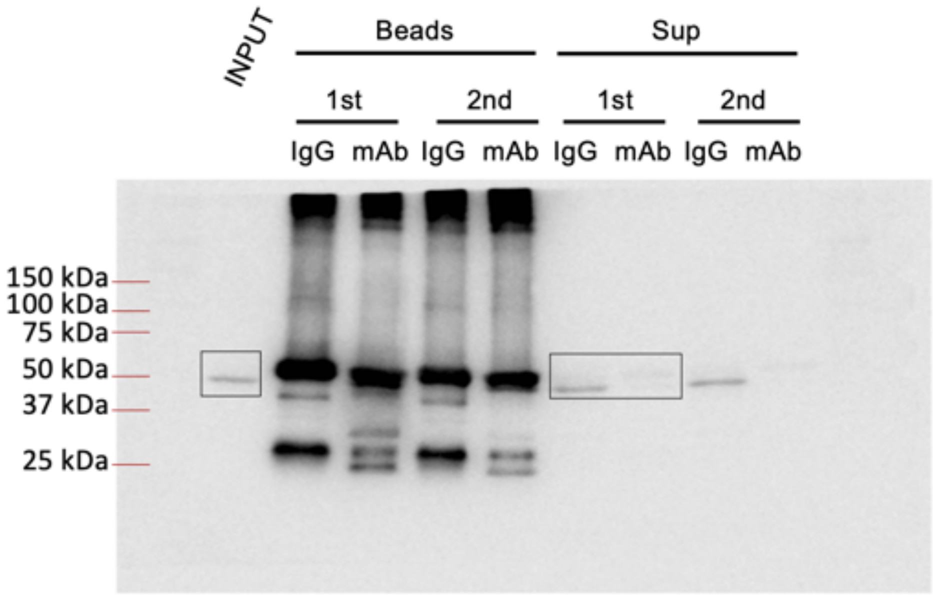

Supplementary Table S1. Antibodies used for immunofluorescence or immunoblotting.

USED FOR	HOST	NAME	SOURCE	IDENTIFIER
primary for immunofluorescence	rat	anti-Ago2 antibody	Millipore	MABE253
primary for western blotting	mouse	anti-Ago2 antibody	Abcam	ab57113
primary for immunofluorescence	mouse	anti-pan-Ago antibody	Millipore	MABE56
primary	mouse	anti-Alix	Cell Signaling Technology	#2171
primary	mouse	anti-Calnexin antibody	BD Biosciences	BD610523
primary	mouse	anti-CD63 antibody (for immunofluorescence analysis)	Abcam	ab193349
primary	rabbit	anti-CD63 antibody (for immunofluorescence analysis)	Abcam	ab252919
primary	mouse	anti-CD63 antibody (for Western blotting analysis)	Novus	NBP2-42225
primary	rabbit	anti-COX IV antibody	Cell Signaling Technology	#4850
primary	rabbit	anti-Dcp1a antibody	Abcam	ab47811
primary	mouse	anti-Flotillin-1 antibody	BD Biosciences	BD610821
primary	goat	anti-Fluorescein antibody	ROCKLAND	600-101-096
primary	mouse	anti-GAPDH antibody	Millipore	MAB374
primary	mouse	anti-GM130 antibody	BD Biosciences	BD610823
primary	mouse	anti-GW182 antibody	Abcam	ab15843
primary	rabbit	anti-JNK1+JNK2+JNK3 antibody	Abcam	ab179461
primary	rabbit	anti-phosphorylated JNK1 (T183) + JNK2 (T183) + JNK3 (T221) antibody	Abcam	ab124956
primary	mouse	anti-Lamp2 antibody	Hybridoma Bank	H4B4
primary	mouse	anti-p230 antibody	BD Biosciences	BD611280
primary	rabbit	anti-p38 MAPK antibody	Cell Signaling Technology	#9212
primary	rabbit	anti-phosphorylated p38 MAPK (T180/Y182) antibody	Cell Signaling Technology	#4511
primary	rabbit	anti-Rab5 antibody	Cell Signaling Technology	#3547
primary	mouse	anti-Rab7 antibody	Cell Signaling Technology	#9367
primary	rabbit	anti-Rab11 antibody	Cell Signaling Technology	#5589
primary	mouse	anti-SLO antibody	Abcam	ab23501
primary	mouse	anti-TSG101 antibody	Novus	NB200-112
primary	rabbit	anti-YBX1 antibody	Abcam	ab76149
secondary	goat	Alexa Fluor 488-conjugated anti-mouse antibody	Invitrogen	#A-11001
secondary	goat	Alexa Fluor 546-conjugated anti-mouse antibody	Invitrogen	#A-11030
secondary	goat	Alexa Fluor 546-conjugated anti-rat antibody	Invitrogen	#A-11081
secondary	goat	Alexa Fluor 647-conjugated anti-rabbit antibody	Invitrogen	#A-21245
secondary	donkey	HRP conjugated anti-goat IgG	Santa Cruz Biotechnology	-
secondary	goat	HRP conjugated anti-mouse IgG	Promega	W402B
secondary	goat	HRP conjugated anti-rabbit IgG	Cell Signaling Technology	#7074
resealing control	mouse	Normal mouse IgG	Santa Cruz Biotechnology	sc-2025
resealing control	rabbit	Normal rabbit IgG	Cell Signaling Technology	#2729

Supplementary Table S2. Primers used for real-time PCR.

USED FOR	NAME	IDENTIFIER or SEQUENCE
SYBR Green assay	18S rRNA_Fw	CGGCTACCACATCCAAGGAA
SYBR Green assay	18S rRNA_Rv	GCTGGAATTACCGCGGCT
TaqMan assay	mmu-miR-34a-5p	mmu481304_mir
TaqMan assay	mmu-miR-122-5p	mmu480899_mir
TaqMan assay	hsa-miR-423-3p	478327_mir
TaqMan assay	cel-miR-39-3p	478293_mir
